# A novel prophage identified in strains from *Salmonella enterica* serovar Enteritidis is a phylogenetic signature of the lineage ST-1974

**DOI:** 10.1099/mgen.0.000161

**Published:** 2018-03-06

**Authors:** Bruno D'Alessandro, Victoria Pérez Escanda, Lucía Balestrazzi, Andrés Iriarte, Derek Pickard, Lucía Yim, José Alejandro Chabalgoity, Laura Betancor

**Affiliations:** ^1^​ Instituto de Higiene, Facultad de Medicina, UDELAR, Montevideo, Uruguay; ^2^​ The Wellcome Trust Sanger Institute, The Wellcome Trust Genome Campus, UK

**Keywords:** *Salmonella*, prophages, lineages

## Abstract

*Salmonella enterica* serovar Enteritidis is a major agent of foodborne diseases worldwide. In Uruguay, this serovar was almost negligible until the mid 1990s but since then it has become the most prevalent. Previously, we characterized a collection of strains isolated from 1988 to 2005 and found that the two oldest strains were the most genetically divergent. In order to further characterize these strains, we sequenced and annotated eight genomes including those of the two oldest isolates. We report on the identification and characterization of a novel 44 kbp *Salmonella* prophage found exclusively in these two genomes. Sequence analysis reveals that the prophage is a mosaic, with homologous regions in different *Salmonella* prophages. It contains 60 coding sequences, including two genes, *gogB* and *sseK3*, involved in virulence and modulation of host immune response. Analysis of serovar Enteritidis genomes available in public databases confirmed that this prophage is absent in most of them, with the exception of a group of 154 genomes. All 154 strains carrying this prophage belong to the same sequence type (ST-1974), suggesting that its acquisition occurred in a common ancestor. We tested this by phylogenetic analysis of 203 genomes representative of the intraserovar diversity. The ST-1974 forms a distinctive monophyletic lineage, and the newly described prophage is a phylogenetic signature of this lineage that could be used as a molecular marker. The phylogenetic analysis also shows that the major ST (ST-11) is polyphyletic and might have given rise to almost all other STs, including ST-1974.

## Data Summary

The raw sequence data of the Uruguayan strains reported in this article are deposited at the European Nucleotide Archive, available at https://www.ebi.ac.uk/ena/data/view/<accession> with the accession numbers ERR036132, ERR036134, ERR036135, ERR036136, ERR036137, ERR036138, ERR036139 and ERR036140 as shown in Table 1.

**Table 1.** *Salmonella* Enteritidis strains isolated in Uruguay used for this work Sources, years of isolation, genome accession numbers and sequence types (STs) are provided, as well as some basic assembly statistics such as number of contigs, N50 and total length of the assembly.

Strain	Source	Year of isolation	Run accession (ebi.ac.uk)	Sample accession (ebi.ac.uk)	ST	Contigs	N50	Total length
31/88	Human (stool)	1988	ERR036132	SAMEA811661	1974	53	376 077	4 711 834
8/89	Human (blood)	1989	ERR036134	SAMEA811659	1974	36	406 197	4 707 919
53/94	Food	1994	ERR036135	SAMEA811657	11	49	406 602	4 705 414
206/99	Food	1999	ERR036136	SAMEA811656	11	40	479 283	4 605 411
251/01	Food	2001	ERR036137	SAMEA811654	11	41	490 253	4 704 527
8/02	Human (stool)	2002	ERR036138	SAMEA811652	11	47	406 188	4 706 958
214/02	Human (blood)	2002	ERR036139	SAMEA811650	11	47	489 353	4 651 049
10/05	Human (stool)	2005	ERR036140	SAMEA811649	11	43	546 223	4 710 596

All the other sequences from *Salmonella enterica* serovar Enteritidis used in the present study are available in EnteroBase (http://enterobase.warwick.ac.uk/species/index/senterica). A summary of the accession codes and metadata of each strain is included in Table S1 (available in the online version of this article).

**Impact Statement**

*Salmonella enterica* serovar Enteritidis is a major serovar isolated from foodborne diseases worldwide. This serovar includes different genetic lineages that could be linked to particular epidemiological behaviours.

In the context of a comparative genomics study, we identified a new prophage found exclusively in a specific genetic lineage of this serovar, the ST-1974. According to the number of available genomes, this lineage is one of the minor lineages in relation to the major sequence type ST-11 that accounts for about 95 % of the total genomes.

This ST-1974 prophage has a mosaic genetic structure and contains two genes that have a known role in *Salmonella* virulence.

Using a collection of *S*. Enteritidis genomes, we state here that the major ST-11 is a diverse and polyphyletic lineage, which gave rise to all the minor ST lineages. We hypothesize that the incorporation of the prophage was determinant to the emergence of the ST-1974 lineage. This finding could help to understand the origins and circulation of minor lineages, which could have impact in epidemiological studies.

## Introduction

*Salmonella* is one of the most important causes of food-borne disease worldwide. The most recent estimates point to non-typhoid *Salmonella* as the pathogen causing the greatest burden of food-borne disease globally, which illustrates its importance for public health [1]. *Salmonella enterica* subspecies *enterica* is a diverse bacterial taxon accounting for about 1600 serovars; however, only a small proportion of them cause the majority of human infections [2]. For example, from 1996 to 2016, the top five *Salmonella* serovars were identified as the cause of 53 % of all confirmed cases in the USA (FoodNet data updated until 2016) [3].

The recent epidemiological history of *Salmonella* infections shows that prevalence of a given serovar is modified over time and geographical location. In the UK, as an example, the prevalent serovar was *Salmonella enterica* serovar Agona until the late 1970s, but then it was replaced by *Salmonella enterica* serovar Typhimurium in the 1980s and again by *Salmonella enterica* serovar Enteritidis in the early 1990s [4, 5].

In Uruguay, *Salmonella enterica* serovar Typhimurium (hereafter *S*. Typhimurium) was the prevalent serovar until 1994, while *Salmonella enterica* serovar Enteritidis (hereafter *S*. Enteritidis) was seldom isolated [6, 7]. A first outbreak of *S*. Enteritidis occurred in 1995, and 2 years later it became the most common serovar in the country [8].

*S*. Enteritidis emerged globally as the prevalent serovar causing salmonellosis in humans in the last decade of the 20th century, remaining nowadays among the most frequent serovar in different regions [3, 9–11].

The great diversity of the *Salmonella* serovars parallels the host adaptability of this genus, and a major factor explaining this diversity is the profusion of mobile elements, phages and prophages present in the genomes of the different serovars [12, 13]. This suggests a great genomic plasticity, which seems to be fundamental to the ecological success of *Salmonella*.

It is known that mobile genetic elements often carry genes that play a fundamental role in bacterial pathogenesis, which in *Salmonella* has been reported extensively [12–15]. Considering that the diversity in prophage cargo can play a role in the epidemiological behaviour or account for differences in the pathogenic potential between serovars or strains, understanding the phage content is important to obtain further insight into the molecular epidemiology of *Salmonella* [12–15].

In previous studies, we characterized phenotypically and genetically a set of *S*. Enteritidis strains that were obtained from different epidemiologic periods in Uruguay, i.e. before, during and after the first *S*. Enteritidis outbreak. Among these strains, the most genetically divergent were the two oldest strains, named 31/88 and 8/89, both isolated more than 7 years before the first *S*. Enteritidis-related food-borne disease outbreak [7]. These two strains were consistently impaired in several virulence-related phenotypic assays, such as motility, epithelial cell invasion, survival in egg albumen and virulence in a chicken model [16]. Using DNA microarrays, we found that the main difference in the genetic content of these two isolates was the presence of several phage-related genes. Among those, we found *gogB*, which so far had never been reported in *S.* Enteritidis and has been implicated in the modulation of the inflammatory response [7].

Here, we obtained the genome sequences of eight *S*. Enteritidis strains including both 31/88 and 8/89 to further analyse those distinct genetic features and found that the main difference is a 44 kbp contig that is present only in these two strains. We mapped this contig to their genomes and characterized it as a novel *Salmonella* prophage. We further studied the presence of this prophage in other *S*. Enteritidis genomes available in public databases, and show that it is a phylogenetic marker of a particular lineage of this serovar.

## Methods

### Bacterial strains, culture and DNA extraction

The isolates were selected from the Uruguayan *S*. Enteritidis previously characterized by genetic and phenotypic methods [7, 16]. Strain names, sources and dates of isolation are shown in Table 1. All strains were kept frozen in LB broth (Sigma-Miller) containing 16 % glycerol (−80 °C) and restored for culture in LB agar (37 °C). Liquid cultures were performed in LB broth at 37 °C with shaking. DNA extraction and purification were performed using commercial purification kits (DNeasy Blood and Tissue Kit, Qiagen).

### Genome sequence processing and bioinformatic analyses

Genome sequencing was performed by Illumina Hiseq2000, as 50–76 bp paired-end runs. For sequence assembly, high-quality reads were selected using sickle (available at https://github.com/najoshi/sickle), and the quality was then checked using FastQC (http://www.bioinformatics.babraham.ac.uk/projects/fastqc/). An average phred score per read of 37 was obtained. On average, a total of 10 722 535 pair-ends reads were produced for each strain with an average insertion size of 321 bp; this means an expected coverage of more than 150×. *De novo* assembly was performed with Spades (version 3.6.1) [17] using a pre-assembly approach with Velvet (version 1.2.10) [18]. The assembly was performed using a range of k-mer sizes between 29 and 127 with a step of 2 and the default parameters in both programs. The genome of strain P125109 (GeneBank ID AM933172.1, NCBI refseq accession NC_011294.1) was used as reference. Contigs that did not match to the reference genome were obtained by using abacas [19] and then searched using NCBI nucleotide blast with the megablast algorithm and the nr database.

Those reference genomes with high identity in the BLASTn results were downloaded and re-annotated in order to perform the comparative genomic analysis. All the genomic sequences were annotated using RASTtk [20], and then analysed using the phast webserver to identify the putative prophage regions [21].

Comparative single-nucleotide polymorphism (SNP) analysis between the prophage found in strains 31/88 and 8/89, and the corresponding contigs from each of the other 154 genomes obtained from EnteroBase was performed using NUCmer (NUCleotide MUMmer version 3.1) and local blast (version 2.2.30).

The comparison figures were generated through BLASTn alignments using EasyFig [22]. The sequence annotation figures were composed with Artemis [23], exported to svg images and edited using Inkscape software (inkscape.org).

### PCR assay

To map the prophage insertion site in the genome of the Uruguayan strains, we designed six primers which hybridize on the extremes of the overlapping contigs to test. The primer sequences are shown in Table 2.

**Table 2.** Primers used in this study, as explained in Methods and Fig. 3

Primer name	Sequence (5′ to 3′)
A	GGCTAACCTGATTCCAACGA
B	CCGGATGTACTCCTGCAAAT
C	GTAGCATCGTGGGATTTTGC
D	CATGTTGCGTTGACGTACAG
E	TGCCAGCGGGAATTAAACCA
F	ATTGGCTTTGTCGGTGTGGA

The final concentrations of reagents for the PCR reaction were as follows: 2 mM MgCl_2_; 0.3 mM dNTPs; 0.3 µM each primer; 2U Taq and 0.2U Pfu DNA polymerases (Thermo Scientific). The cycling programme used was a first step of 3 min at 95 °C, then 30 cycles of 30 s at 95 °C, 30 s at 53 °C and 1 min 30 s at 72 °C, with a final step of 5 min at 72 °C. PCR products were visualized by agarose gel electrophoresis, using 2.0 % agarose gel stained with Gelgreen (Biotium).

### Multilocus sequence typing and phylogenetic analysis

We submitted the fastq files with their associated metadata to the EnteroBase, which performs a pipeline of assembly, annotation and several *in silico* typing schemes, including Achtman 7 gene multilocus sequence typing (MLST) and ribosomal MLST (rMLST) (http://enterobase.warwick.ac.uk).

For the selection of the genomes to include in the phylogenetic analysis, we obtained the total list of rMLST representatives (provided by EnteroBase in the public workspace named ‘rST representatives’) and selected those genomes predicted to be *S.* Enteritidis and belonging to the group eBG4, as this is the main eBURST group (eBG) for this serovar [24, 25]. This selection criterion allowed the inclusion of a wide range of genomic variants from this serovar, with a minimized redundancy of taxonomic units. The final dataset for phylogenetic analysis comprised 171 genomes selected from Enterobase, 22 genomes representative of the major clusters reported in a previous *S*. Enteritidis phylogenetic study [26] and the eight *S*. Enteritidis genomes reported here. P125109 was again used as the reference genome, and the *Salmonella enterica* serovar Gallinarum strain 287/91 as the outgroup (accession NC_011274.1). We obtained a SNP alignment for these 203 genomes using the EnteroBase SNP tree tool, and selecting the genome of strain P125109 as reference. This tool uses last software to obtain SNPs from the assembled genomes and filter out repetitive regions and low-quality variants as detailed by Zhou *et al.* [24]. We used the default setting that considers only those SNPs present in at least 95 % of the genomes. The SNP matrix obtained from EnteroBase was used as input for further phylogenetic analysis using the maximum-likelihood method with GTR Gamma model as implemented in RAxML v8.2.9 [27]. The rapid bootstrap test was used to evaluate node support [28]. The ascertainment bias correction option was applied as recommended by the authors for SNPs (and other data matrices including only variable features). Gaps were completely removed from the alignment.

In order to assess the robustness of the phylogeny, we also performed a Bayesian analysis using beast software (version 2.4.7) with the parameters obtained from a previous phylogenetic study [26, 29]. Briefly, we ran four independent chains of length 30 million, and one chain of length 10 million, using a clock rate of 2.2×10^−7^ substitution sites per year, a relaxed lognormal molecular clock and a constant effective population size. The evolutionary model GTR was selected after running jModelTest (version 2.1.10) using the SNP alignments as input [30].

## Results and Discussion

### Identification of a novel *S.* Enteritidis prophage

When compared with the reference genome, a 44 kbp contig was identified as present exclusively in the genome of the two pre-epidemic strains 31/88 and 8/89. A nucleotide megablast search of the NCBI nr collection database revealed that this contig does not share homology with any *S.* Enteritidis genome from this database. The same result was obtained after a local blastn search to the other six *S.* Enteritidis genomes here reported. However, it displayed high nucleotide identity (>98 %) with some *S*. Typhimurium genomic regions. We selected *S*. Typhimurium reference strains SL1344, DT104 and USDA-ARS-USMARC-1880 as representatives to perform further comparative studies (NCBI GenBank accessions FQ312003.1, HF937208.1 and NZ_CP014981.1, respectively).

**Fig. 1.**
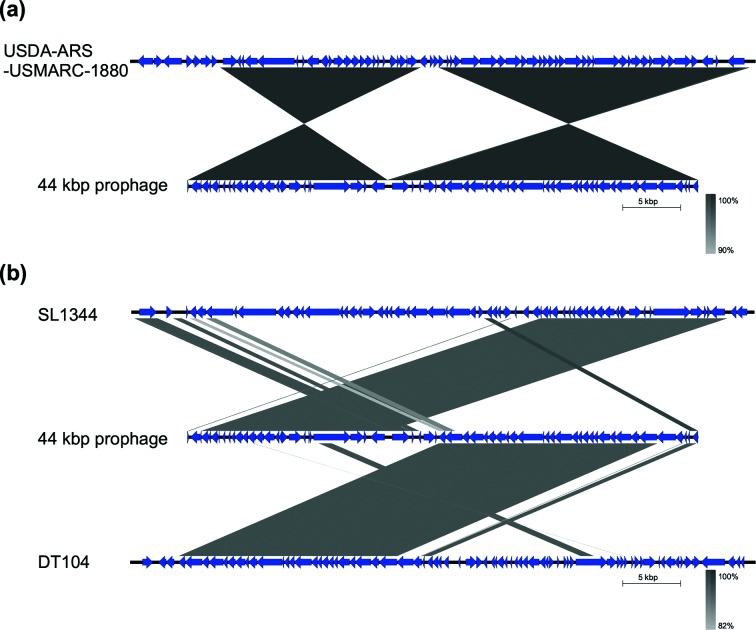
(a) Nucleotide identity between the 44 kbp contig (bottom) and the corresponding genomic region in *S*. Typhimurium strain USDA-ARS-USMARC-1880 (top). (b) Nucleotide identity between the prophage present in the genome of *S*. Thypimurium strain SL1344 (top), the ST-1974 prophage (middle) and the genomic region predicted as a prophage by phast in strain DT104 (bottom). The nucleotide identity of the homologous regions (percentage) is indicated by a gradient from white to grey, shown at the bottom right of each panel. The 5 kbp bar indicates the scale of the sequence length.

The entire contig sequence is highly similar to one specific region (position 1 307 784 to 1 353 495) in strain USDA-ARS-USMARC-1880, but not as a continuous alignment. Instead, the alignment is separated in two halves, each one with an inverted counterpart in the genome of the USDA-ARS-USMARC-1880 strain (Fig. 1). Similarly, the 5′ half of the prophage (position 1152 to 170 284) is almost identical to a region in the genome of SL1344 (position 2 761 354 to 2 777 486), while the 3′ half (position 21 708–40 578) matches with a different region in the genome of DT104 (1 954 192 to 1 973 076) (Fig. 1).

**Fig. 2.**
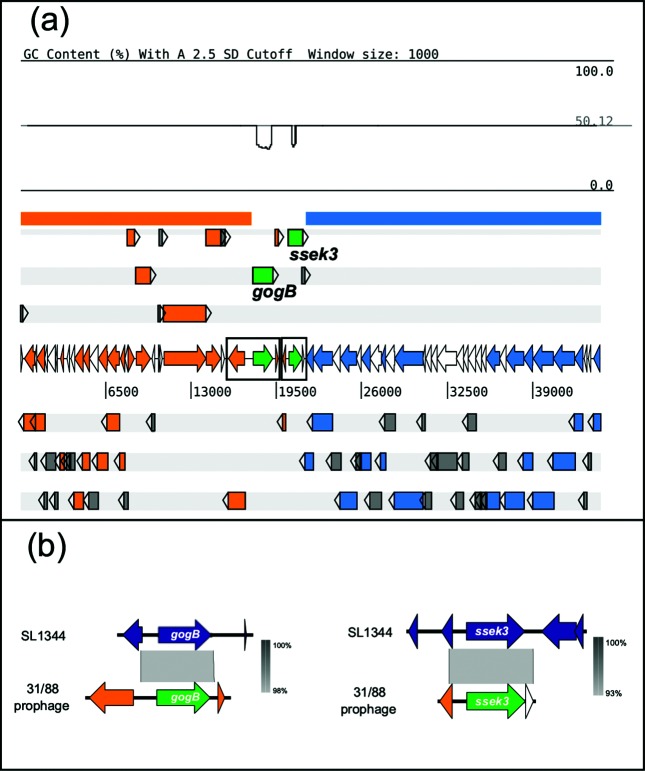
(a) Modular organization of the ST-1974 prophage and the presence of cargo genes. At the top, a DNA G+C content graph is shown (percentage G+C with two standard deviation cutoff, as generated by Artemis). Marked in orange, is the region enriched in regulatory or replication phage functions. The region marked in blue is enriched in structural phage functions. Individual coding sequences are represented by arrows on the six coding frames, with colours corresponding to functions. Moron genes, *gogB* and *sseK3*, are indicated in green. Those genes encoding hypothetical proteins are marked in grey. Annotation details are shown in Table S2. (b) The alignment between the moron genes from strains 31/88 and SL1344 is shown. The SL1344 genetic regions include 1 kb upstream and downstream of the *gogB* and *sseK3* coding sequences.

We found that the region in strain SL1344 is part of a prophage (named SLP272/Gifsy-1), while the regions in USDA-ARS-USMARC-1880 and DT104 were not annotated as prophages. Using phast to predict unidentified prophages, we found that all the regions of high similarity to the 44 kbp contig were contained in predicted prophages (Table S2 and Fig. 1). Furthermore, the 44 kbp contig itself was predicted by phast as an intact prophage region (Fig. 2a). This strongly suggests that the contig encodes a prophage sequence (designated ST-1974 prophage) that is likely a mosaic based on the alignment observed with the *S*. Typhimurium strains. Following this hypothesis, we devised a strategy to map the location of the putative prophage on the chromosome of the strains.

### Genome context of the ST-1974 prophage in *S*. Enteritidis strains

**Fig. 3.**
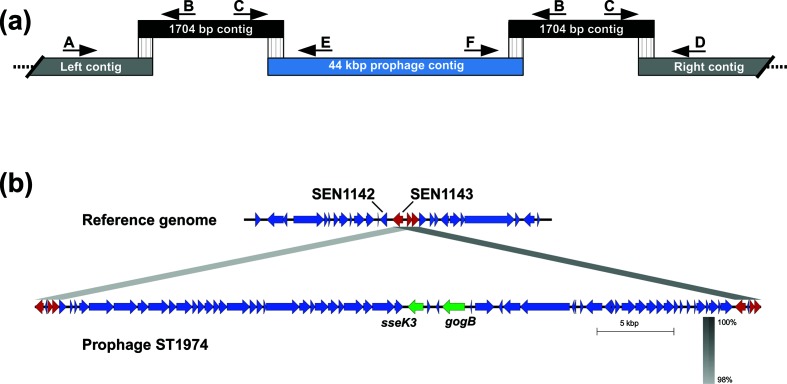
Location of the prophage in the chromosome of strains 31/88 and 8/89. (a) A proposed order for the 44 kbp contig (light blue) in the chromosome with the 1704 bp contigs (in black) flanked by right and left contigs (in grey) is shown. Arrows labelled with letters indicate the PCR primers designed to amplify the corresponding regions in strains with or without the prophage. DNA from strains 31/88 and 8/89 yielded products only from the combinations of primers corresponding to the proposed assembly (primer combinations AB, CE, FB and CD). Conversely, PCR using DNA from strains P125109 or 8/02 yielded products as expected for the sequence without the prophage (primer combinations AB and CD). (b) Alignment between the reference genome and the 31/88 genome harbouring the ST-1974 prophage is shown. Arrows representing the genes within the 1704 bp region are marked in red. The homologous region corresponds to the 1704 bp. Here, the ST-1974 prophage was reversed with respect to the orientation shown in Figs 1 and 2, to match the gene ordering of the reference chromosome. The percentage sequence identity is scaled from light to dark grey.

First, we searched for sequence overlap between the 44 kpb contig and the other contigs in the draft genomes carrying the ST-1974 prophage. We found only one small contig (1704 bp) that overlapped with both ends of the 44 kpb sequence (Fig. 3a). Also, each end of the same small contig overlapped with a different contig of the draft genome (indicated as left and right contigs in Fig. 3a). Assuming that the prophage is integrated into the chromosome, we hypothesized that the prophage should be flanked by two copies as direct repeats of the 1704 bp sequence. In order to confirm that this organization was correct, we designed a PCR assay with primers matching the ends of each of these contigs (Fig. 3a).

The PCR results confirmed the location of the prophage in the chromosome as evidenced by the flanking directly repeated sequence of 1704 bp, the signature of the recombination mechanism of insertion. The sequence analysis showed that in comparison with the reference genome, the prophage is inserted between genes SEN1142 and SEN1143, in the position 1 235 779 as shown in Fig. 3(b). Furthermore, the genetic region in which the prophage is inserted is annotated in the P125109 genome as a remnant prophage (named phi-SE12) as it contains some genes related to the Gifsy-2 phage (e.g. *sodCI*, *gogA*, *ompX* and *sopE*) [31]. This suggests that this locus may have a history of phage insertions/deletions in the course of evolution.

### Characterization of the ST-1974 prophage gene content

The 44 kbp prophage contig was annotated using RASTtk, and as a result 60 coding sequences were predicted. Most of the annotated coding sequences were phage-related proteins (with structural, regulatory or replicative functions represented) as well as some hypothetical phage genes (Table S3).

Interestingly, while structural genes were located within the 5′ region (homologous to DT104), regulation and replication genes were exclusively found in the 3' region (homologous to SL1344) (Fig. 2a and Table S3). Similar organization of genes in functional modules has been described extensively in phage genomes as a result of a process named modular exchange, a driving force in the evolution of phages [12]. In the modular exchange model, segments of DNA from one phage are replaced by recombination with segments of a different phage, usually with genes of similar function to those in the segment replaced, giving rise to a new phage. Therefore, our observations suggest that this prophage originated as a result of the modular exchange process.

Interestingly, the 44 kbp prophage contains the genes *gogB and sseK3*, which could be recognized as *moron* genes, namely carried by phages but not related to its life cycle. These genes are transcribed independently, and their presence usually provides a competitive advantage to the bacteria, e.g. an increase of its virulence potential [12, 32]. Morons often have a different DNA G+C content than the surrounding phage sequence, and that is what we found for these two genes (Fig. 2a).

Both moron genes encode for type III secretion system (T3SS) effectors, which were reported to act in inhibiting the activation of NF-κB [33, 34].

GogB was first described in the phage Gifsy-1 of *S*. Typhimurium strains LT2 and SL1344 [14, 35]. It has been established that GogB acts as an anti-inflammatory effector when the bacteria encounter the host cell by inhibiting the activation of NF-κB, playing a central role in limiting host tissue damage in a model of chronic infection [33, 36]. Using a *Salmonella* pangenome microarray, we have already reported the presence of *gogB* in strains 8/89 and 31/88 [7], but now we describe the genomic context of *gogB* and its location within a prophage acquired by these strains. Here, we compared the region encoding the *gogB* gene in the ST-1974 prophage with the *gogB* region in the genome of *S.* Typhimurium SL1344, where this protein was﻿ characterized functionally [35]. The alignment showed 98 % identity between the two proteins and 99 % nucleotide identity (coverage 100 %). In addition, the comparison of the genomic regions (1 kb upstream and 1 kb downstream of the gene) showed an extended region of 98 % identity comprising the gene and about 500 bp upstream (Fig. 2b).

Similar to *gogB*, *sseK3* encodes a protein that modulates inflammatory response once the bacteria encounter the host cell [37]. Its presence was first reported in prophages ST64B and SE20 from *S.* Typhimurium SL1344 and *S*. Enteritidis P125109, respectively [31, 37]. Recent studies showed that SseK3 has a similar effect in the host cell to its homologue NleB, present in enteropathogenic and enterohaemorrhagic *Escherichia coli* [38, 39]. However, SseK3 interacts with different host proteins than NleB, and inhibits the TNF-alpha-induced NF-kappaB activation by interaction with host proteins not yet determined [34, 38]. Similarly to *gogB*, we compared the genomic region containing the *ssek3* gene from SL1344 with the ST-1974 prophage. The alignment showed 95 % nucleotide and amino acid identity with a coverage of 100 %. In addition, the comparison of an extended region showed high nucleotide identity (93 %), including in the upstream sequence (Fig. 2b).

These results show that the two moron genes and their promoter regions are highly similar to those harboured by SL1344, where these genes are functional [31, 35]. Further, in SL1344, both genes are expressed and secreted through a SPI2-encoded T3SS [35, 37]. Given that the *S.* Enteritidis SPI2 T3SS is functional, it is tempting to speculate that both genes may retain their biological functions in *S.* Enteritidis. Further studies are required to evaluate if expression of these genes has a similar impact on the biology of these strains to that in *S*. Typhimurium.

The apparent redundancy of having multiple effectors affecting the same host pathway is thought to be a mechanism evolved for fine tuning the host's inflammatory response in order to benefit bacterial colonization. This type of modulation is important for the establishment of the infection, as the pathogen would manipulate both enhancers and dampers of the inflammation in order to control bacterial density in the gut [36, 40].

### The novel 44 kbp prophage is a phylogenetic marker for the ST-1974 lineage

Strains 31/88 and 8/89 were *in silico* MLST typed and both were determined to be sequence type (ST) 1974 (ST-1974). The ST-1974 differs from the main ST-11 *S*. Enteritidis ST by a single nucleotide substitution in the *hemD* allele among the seven genes included in the Achtman scheme [25]. It should be noted that about 95 % of the *S*. Enteritidis genomes present in EnteroBase belong to ST-11 (more than 19 000 in total) [41].

Even though we could not find any *S*. Enteritidis genomes carrying the ST-1974 prophage in the NCBI nr database, at the time of writing this article, 154 *S*. Enteritidis genomes typed as ST-1974 were present in EnteroBase (Table S4). This ST accounted for less than 1 % of the *S*. Enteritidis genomes but is the third most common in EnteroBase. The analysis of available metadata for these genomes showed that these strains were isolated evenly between 2003 and 2017, indicating that the ST-1974 lineage is currently circulating in America. Most of the strains (106 out of 154) were isolated from human infections (86 from stool, 12 from blood and 8 from other sites of infection), demonstrating that ST-1974 strains are able to cause different types of infections in humans. All 14 non-human strains were isolated from food, 13 of which were obtained from vegetable sources. There is no available data for the remaining 34 strains (Table S4).

We analysed by local blastn the 154 ST-1974 genomes and found that the 44 kbp prophage is present in all of them. The results show an identity of at least 99.97 % with a coverage of 99 %, evidencing that the prophage is highly conserved among ST-1974 genomes. A total of 30 SNPs are distributed among 152 genomes, with a maximum of 10 SNPs in a single genome and maximum of 16 genomes per SNP. Only one strain presents a SNP that introduces a premature stop codon in the CDS-50; therefore, producing a hypothetically disrupted gene (Fig. S1). As revealed by these results, the novel prophage has a very low variability, with an almost invariable genetic content among the ST-1974 genomes.

To perform further comparative and phylogenetic analysis, we used a selection of 203 genomes including representatives of all *S*. Enteritidis STs belonging to eBG4 from EnteroBase and strains selected from the five major lineages reported by Deng and colleagues for this serovar [26]. We searched for the phage in this selection of genomes and found that it is only present in ST-1974 genomes. Therefore, we hypothesized that this prophage could represent a phylogenetic marker for the ST-1974 lineage.

**Fig. 4.**
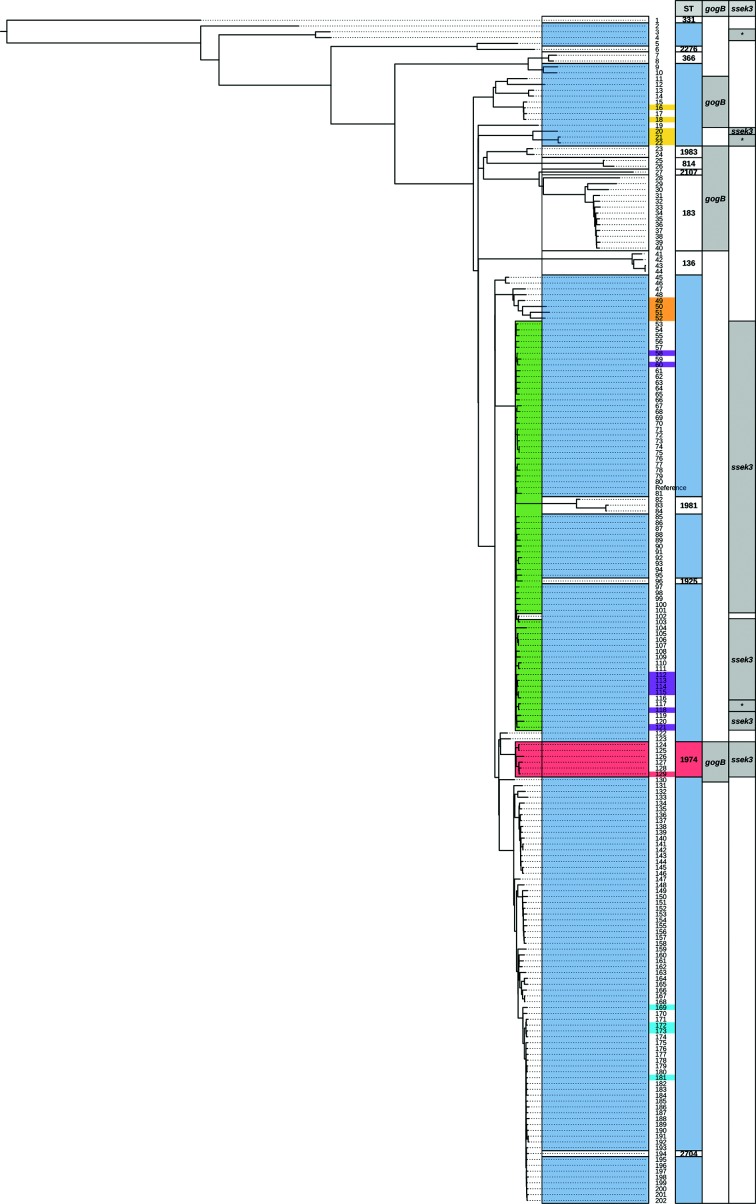
Phylogenetic tree of representative *S*. Enteritidis genomes. The tree includes 203 genomes representative of *S*. Enteritidis using the genome of P125109 as reference. The genome of *S*. Gallinarum strain 287/91 was used as an outgroup and to re-root the tree. Numbers at the tips of the leaves represent genomes as numbered in Table S1. Highlighted leaf tips with different colours indicate each of the five lineages reported by Deng *et al.* [26]. In yellow, lineage I; in orange, lineage II, in violet, lineage III; in light red, lineage IV (ST-1974 lineage); and in sky blue, lineage V. The clusters highlighted at the very left of the leaves are those that harbour the prophages SE20 (in green) or ST-1974 (in red). The first column to the right of the tree indicates the MLST ST number, while the major ST-11 is higlighted in light blue. The second and third columns indicate the presence of *gogB* or *sseK3* genes. (*) indicates those genomes with a hypothetically disrupted *ssek3* gene. Fig. S2 shows a version of this tree with the bootstrap support values for the branching.

In order to infer the phylogenetic relatedness of this lineage in the broad context of *S*. Enteritidis, we reconstructed a whole-genome phylogenetic tree. The SNP tree obtained using the maximum-likelihood method shows that all ST-1974 strains are clustered together as a monophyletic group (Fig. 4). Similarly, the other STs represented in the tree also are monophyletic lineages, with the exception of ST-11, which is polyphyletic. This phylogenetic distribution suggests that all minor STs diverged from an ST-11 ancestor. We also performed a Bayesian analysis obtaining highly similar results, confirming the robustness of the proposed phylogeny.

Achtman *et al.* have already suggested that ST-11 was the central ST for all the eBG4 STs, and our results strongly support their observation [25].

The *S*. Enteritidis phylogeny obtained in our study is in agreement with the one proposed by Deng and colleagues, as our phylogeny distinguished all the lineages that were reported previously [26] (Figs 4 and S2). Furthermore, our results give better support to those lineages and, interestingly, the lineage that was represented by only one strain in Deng's work is recognized here as ST-1974. As our selection of genomes included more representatives of the *S*. Enteritidis diversity, we were able to identify several other lineages that correspond to different STs (Fig. 4).

The phylogenetic tree also suggests that the ST-1974 prophage was incorporated in the most recent common ancestor of this lineage.

As pointed out above, *gogB* was detected previously in *S.* Enteritidis strains 8/89 and 31/88, and now we demonstrate that the gene is carried by the ST-1974 prophage. Furthermore, we looked for its presence in all the *S.* Enteritidis genomes from our phylogenetic analysis. As a result, we confirmed that 34 of 203 genomes contain this gene, including the four representatives of ST-1974 and others from different STs (see Fig. 4). We analysed the genetic context of *gogB* in the non-ST-1974 genomes and confirmed that it is always present in regions predicted as prophages by phast (results not shown).

We also looked for the presence of *sseK3,* the other moron gene found in the novel prophage, in the other genomes of the phylogeny. As expected, all strains carrying SE20 or ST-1974 prophages also carry *ssek3* (Fig. 4). The presence of *sseK3* is strongly associated with these prophages, as only one genome carrying *ssek3* has neither SE20 nor the ST-1974 prophage (i.e. strain 81 748 identified by number 20 in Fig. 4). However, the co-occurrence of *gogB* and *ssek3* is only seen in the ST-1974 lineage.

Several reports provide evidence for the idea that *S*. Enteritidis lineages are strongly associated with differential prophage cargo [14, 42, 43]. A recent report showed the emergence of two phylogenetic clades, composed exclusively of strains from sub-Saharan Africa with a marked epidemic profile of invasive disease [42]. The authors also distinguished another two main clades with a marked gastroenteritis epidemic profile. Each of those four clades harbours a clear differential phage cargo, as well as other accessory genes mostly associated with horizontal acquisition. The work of Feasey *et al.* suggests that diversification events occurring after acquisition of new prophages could be a step in the establishment of a new epidemiological course [42]. In the same way, here we observed that the ST-1974 lineage also appears to have diversified after the acquisition of a new prophage.

According to the available metadata for the ST-1974 strains, they were isolated from distant geographic regions of the American continent from 1988 to 2017 (Table S4). The vast majority of those strains were isolated from the USA, with six from the Dominican Republic plus the two from Uruguay, evidencing that ST-1974 is broadly spread in America where it coexists with the main ST-11 lineage. Still, the number of available ST-1974 genomes is remarkably smaller than that for ST-11.

We report here that strains from this lineage were isolated from different types of human infections as well as food sources, oddly enough almost always vegetables. However, the available information is not sufficient to understand why ST-1974 conforms a minor lineage. It is possible to hypothesize that this could be related to their presence in different environmental niches or due to a very low frequency as food contaminant compared with ST-11. Further studies searching for *S*. Enteritidis ST-1974 in different environments would be helpful to elucidate these questions, and the use of the prophage sequence as a molecular marker for this lineage should be considered. Knowledge of the environmental niches and epidemiology of minor lineages other than the prevalent ST-11 would certainly be helpful to improve the epidemiological surveillance of *Salmonella*.

## Data bibliography

1. D'Alessandro B, Pérez Escanda V, Balestrazzi L, Irirate A, Pickard D, Yim L, Chabalgoity JA, Betancor L. European Nucleotide Archive. ERR036132, ERR036134, ERR036135, ERR036136, ERR036137, ERR036138, ERR036139, ERR036140 (2018).

## Supplementary Data

430 Supplementary File 1
