# Supplementary material for: A novel prophage identified in strains from Salmonella enterica serovar Enteritidis is a phylogenetic signature of the lineage ST-1974
Source: Microb Genom. 2018 Mar 6;4(3):e000161. doi: 10.1099/mgen.0.000161 (PMC5885013; doi:10.1099/mgen.0.000161)
Supplement: Supplementary File 1 [file mgen-4-161-s001.pdf]

Table S1: Metadata for the 203 *S.* Enteritidis genomes used in the phylogeny, including the reference.

					Achtman allele sequence type																				
**number_ID**	**Name in EnteroBase**	**ST**	**eBG**	**Serotype (Predicted SISTR)**	**aroC**	**dnaN**	**hemD**	**hisD**	**purE**	**sucA**	**thrA**	**EnteroBase Uberstrain**	**Source Niche**	**Source Type**	**Source Details**	**Collection Year**	**Continent**	**Country**	**Region**	**City**	**Lab Contact**	**Bio Project ID**	**Project ID**	**Sample ID**	**Secondary Sample ID**
1	287/91 / NCTC 13346	331	4	Gallinarum	122	2	42	7	31	6	11	SAL_EA9782AA	Poultry	Avian	Chicken	1991	South America	Brazil			European Bioinformatics Institute	PRJEA30689		SAMEA1705927	ERS000073
2	NY-swgs1565	11	4	Enteritidis	5	2	3	7	6	6	11	SAL_BA1134AA	Human	Human	Other; Homo sapiens	2014	North America	United States	New York	New York	NY State Dept. of Health, Wadsworth Center	PRJNA227458	SRP033012	SAMN03272680	SRS801674
3	NY-swgs1521	11	4	Enteritidis	5	2	3	7	6	6	11	SAL_DA1703AA	Human	Human	stool; Homo sapiens	2014	North America	United States	New York	New York	NY State Dept. of Health, Wadsworth Center	PRJNA227458	SRP033012	SAMN03142768	SRS734917
4	NY-swgs1299	11	4	Enteritidis	5	2	3	7	6	6	11	SAL_DA6263AA	Human	Human	Stool; Homo sapiens	2014	North America	United States	New York	New York	NY State Dept. of Health, Wadsworth Center	PRJNA227458	SRP033012	SAMN02851127	SRS643423
5	34_61-sc-2013-06-10T14:58:16Z-1611356	11	4	Enteritidis	5	2	3	7	6	6	11	SAL_BA0147AA				1961					The Wellcome Trust Sanger Institute	PRJEB634	ERP001965	SAMEA2078333	ERS255532
6	NY57039180	2276	4	Enteritidis	523	2	3	7	6	6	11	SAL_AA7192AA	Human	Human	Stool/Fecal; Homo sapiens	2015	North America	United States	New York	New York	NY State Dept. of Health, Wadsworth Center	PRJNA227458	SRP033012	SAMN03892551	SRS1017016
7	261627-sc-2013-04-16T07:32:25Z-1611057	366	4	Enteritidis	5	2	3	7	125	6	11	SAL_BA4041AA				2008					The Wellcome Trust Sanger Institute	PRJEB634	ERP001965	SAMEA2062724	ERS235877
8	543-sc-2012-11-09T12:21:02Z-1500144	366	4	Enteritidis	5	2	3	7	125	6	11	SAL_BA5234AA									The Wellcome Trust Sanger Institute	PRJEB634	ERP001965	SAMEA1712443	ERS188208
9	D35807-sc-2011-03-31T14:07:56Z-10447	11	4	Enteritidis	5	2	3	7	6	6	11	SAL_DA7454AA				2005					The Wellcome Trust Sanger Institute	PRJEB2478	ERP000596	SAMEA698479	ERS026076
10	3493-sc-2012-11-09T12:20:58Z-1500140	11	4	Enteritidis	5	2	3	7	6	6	11	SAL_BA5391AA									The Wellcome Trust Sanger Institute	PRJEB634	ERP001965	SAMEA1712495	ERS188204
11	M204 / NCTC20341	11	4	Enteritidis	5	2	3	7	6	6	11	SAL_BA9151AA				1938					THE SANGER CENTER	PRJEB3255	ERP001784	SAMEA2055714	ERS222783
12	20_55-sc-2013-06-10T14:58:03Z-1611345	11	4	Enteritidis	5	2	3	7	6	6	11	SAL_BA0192AA				1955					The Wellcome Trust Sanger Institute	PRJEB634	ERP001965	SAMEA2078105	ERS255521
13	P177-sc-2013-05-22T09:57:00Z-1611210	11	4	Enteritidis	5	2	3	7	6	6	11	SAL_BA0083AA				2006					The Wellcome Trust Sanger Institute	PRJEB634	ERP001965	SAMEA2074345	ERS248543
14	46132	11	4	Enteritidis	5	2	3	7	6	6	11	SAL_CA6705AA	Human	Human	Human; Homo sapiens	2014	Europe	United Kingdom	England	London	Gastrointestinal Bacteria Reference Unit, Public Health England	PRJNA248792	SRP042645	SAMN03465765	SRS897902
15	D43980-sc-2012-11-09T12:21:34Z-1500178	11	4	Enteritidis	5	2	3	7	6	6	11	SAL_BA5648AA				2007					The Wellcome Trust Sanger Institute	PRJEB634	ERP001965	SAMEA1712250	ERS188242
16	2009K-0479	11	4	Enteritidis	5	2	3	7	6	6	11	SAL_GA3046AA	Human	Human	homo sapiens	2008 (*)	Africa	Uganda			The Genome Center at Washington University School of Medicine in St. Louis	PRJNA168746	SRP044858	SAMN02941052	SRS666482
17	35773-sc-2012-11-09T12:24:53Z-1500387	11	4	Enteritidis	5	2	3	7	6	6	11	SAL_BA5244AA				2011					The Wellcome Trust Sanger Institute	PRJEB634	ERP001965	SAMEA1712456	ERS188451
18	2009K-0477	11	4	Enteritidis	5	2	3	7	6	6	11	SAL_GA3047AA	Human	Human	homo sapiens	2008 (*)	Africa	Uganda			The Genome Center at Washington University School of Medicine in St. Louis	PRJNA168745	SRP044857	SAMN02941051	SRS666481
19	9_63-sc-2013-06-10T14:58:18Z-1611358	11	4	Enteritidis	5	2	3	7	6	6	11	SAL_BA0149AA				1963					The Wellcome Trust Sanger Institute	PRJEB634	ERP001965	SAMEA2080572	ERS255534
20	81748	11	4	Enteritidis	5	2	3	7	6	6	11	SAL_GA2997AA	Wild Animal	Marsupial	Diagnostic; Wild mammal	2005	North America	United States			The Genome Center at Washington University School of Medicine in St. Louis	PRJNA168793	SRP044980	SAMN03784391	SRS674607
21	04-0307	11	4	Enteritidis	5	2	3	7	6	6	11	SAL_GA3048AA	Environment	ND	almond processing plant	2004	North America	United States	Arizona		The Genome Center at Washington University School of Medicine in St. Louis	PRJNA168744	SRP044856	SAMN02941050	SRS666479
22	J0903	11	4	Enteritidis	5	2	3	7	6	6	11	SAL_GA3013AA	Environment	Soil/Dust	drag swab	2001	North America	United States	California		The Genome Center at Washington University School of Medicine in St. Louis	PRJNA168762	SRP044927	SAMN03784375	SRS674591
23	A59130-sc-2012-11-09T12:25:17Z-1500411	1983	4	Enteritidis	5	2	3	591	6	6	11	SAL_BA5173AA				2010					THE SANGER CENTER	PRJEB634	ERP001965	SAMEA1712377	ERS188475
24	D23071-sc-2011-03-31T14:07:56Z-10446	1983	4	Enteritidis	5	2	3	591	6	6	11	SAL_DA7453AA				2003					The Wellcome Trust Sanger Institute	PRJEB2478	ERP000596	SAMEA698478	ERS026075
25	M133 / NCTC20058	814	4	Enteritidis	233	2	3	7	6	6	11	SAL_BA5848AA				1938					The Wellcome Trust Sanger Institute	PRJEB3255	ERP001784	SAMEA1711466	ERS179694
26	H121440322	814	4	Enteritidis	233	2	3	7	6	6	11	SAL_DA1151AA	Human	Human	Human; Homo sapiens	2012	Europe	United Kingdom	England	London	Gastrointestinal Bacteria Reference Unit, Public Health England	PRJNA248792	SRP042645	SAMN03168724	SRS740014
27	6_66-sc-2013-06-10T14:58:23Z-1611363	2107	4	Enteritidis	5	460	3	7	60	6	11	SAL_BA0180AA				1966					The Wellcome Trust Sanger Institute	PRJEB634	ERP001965	SAMEA1971130	ERS255539
28	FNW18t74	183	4	Enteritidis	5	2	3	7	60	6	11	SAL_DA7160AA	Aquatic	Fish	yellow croaker, frozen	2008	Asia	China			Food and Drug Administration - Center for Food Safety and Applied Nutrition (US)	PRJNA186035	SRP018785	SAMN02678735	SRS614122
29	18_62-sc-2013-06-10T14:58:17Z-1611357	183	4	Enteritidis	5	2	3	7	60	6	11	SAL_BA0168AA				1962					The Wellcome Trust Sanger Institute	PRJEB634	ERP001965	SAMEA2078334	ERS255533
30	1_59-sc-2013-06-10T14:58:10Z-1611351	183	4	Enteritidis	5	2	3	7	60	6	11	SAL_BA0191AA				1959					The Wellcome Trust Sanger Institute	PRJEB634	ERP001965	SAMEA2078109	ERS255527
31	ASERH085580-sc-2013-02-28T11:35:29Z-1570957	183	4	Enteritidis	5	2	3	7	60	6	11	SAL_EA0995AA									The Wellcome Trust Sanger Institute	PRJEB1271	ERP002103	SAMEA1920618	ERS218116
32	H123480474	183	4	Enteritidis	5	2	3	7	60	6	11	SAL_DA0881AA	Human	Human	Human; Homo sapiens	2012	Europe	United Kingdom	England		Gastrointestinal Bacteria Reference Unit, Public Health England	PRJNA248792	SRP042645	SAMN03169050	SRS740436
33	99-02302	183	4	Enteritidis	5	2	3	7	60	6	11	SAL_AA9668AA				1999					The Wellcome Trust Sanger Institute	PRJEB2289	ERP000348	SAMEA2342569	ERS400254
34	ASERH122277-sc-2013-02-28T11:35:08Z-1570940	183	4	Enteritidis	5	2	3	7	60	6	11	SAL_EA1012AA									The Wellcome Trust Sanger Institute	PRJEB1271	ERP002103	SAMEA1920603	ERS218099
35	ASERH110064-sc-2013-02-28T11:34:50Z-1570925	183	4	Enteritidis	5	2	3	7	60	6	11	SAL_EA0954AA									The Wellcome Trust Sanger Institute	PRJEB1271	ERP002103	SAMEA1920587	ERS218084
36	65321	183	4	Enteritidis	5	2	3	7	60	6	11	SAL_CA4034AA	Human	Human	Human; Homo sapiens	2014	Europe	United Kingdom	England		Gastrointestinal Bacteria Reference Unit, Public Health England	PRJNA248792	SRP042645	SAMN03476015	SRS902571
37	44686	183	4	Enteritidis	5	2	3	7	60	6	11	SAL_CA6826AA	Human	Human	Human; Homo sapiens	2014	Europe	United Kingdom	England		Gastrointestinal Bacteria Reference Unit, Public Health England	PRJNA248792	SRP042645	SAMN03465637	SRS898020
38	82515	183	4	Enteritidis	5	2	3	7	60	6	11	SAL_EA7164AA	Human	Human	Human; Homo sapiens		Europe	United Kingdom			Public Health England	PRJNA248792	SRP042645	SAMN04363909	SRS1220238
39	60067	183	4	Enteritidis	5	2	3	7	60	6	11	SAL_BA7691AA	ND	Other Mammal	ND	2014	Europe	United Kingdom		London	Public Health England - Gastrointestinal Bacteria Reference Unit	PRJNA248792	SRP042645	SAMN03480257	SRS906752
40	40797	183	4	Enteritidis	5	2	3	7	60	6	11	SAL_CA4296AA	Human	Human	Human; Homo sapiens	2014	Europe	United Kingdom			Gastrointestinal Bacteria Reference Unit, Public Health England	PRJNA248792	SRP042645	SAMN03475752	SRS902465
41	M6 / NCTC20017	136	4	Enteritidis	5	2	3	61	6	6	11	SAL_BA5854AA				1917					The Wellcome Trust Sanger Institute	PRJEB3255	ERP001784	SAMEA1711410	ERS179690
42	ST136	136	4	Enteritidis	5	2	3	61	6	6	11	SAL_AA9086AA									The Wellcome Trust Sanger Institute	PRJEB634	ERP001965	SAMEA1930130	ERS330784
43	ST136	136	4	Enteritidis	5	2	3	61	6	6	11	SAL_AA9084AA									The Wellcome Trust Sanger Institute	PRJEB634	ERP001965	SAMEA1930132	ERS330786
44	10_63-sc-2013-06-10T14:58:19Z-1611359	136	4	Enteritidis	5	2	3	61	6	6	11	SAL_BA0161AA				1963					The Wellcome Trust Sanger Institute	PRJEB634	ERP001965	SAMEA2078335	ERS255535
45	CFSAN031663	11	4	Enteritidis	5	2	3	7	6	6	11	SAL_EA9102AA	Livestock	Swine	feces; swine	2005	Africa	Ethiopia			FDA Center for Food Safety and Applied Nutrition	PRJNA275961	SRP057873	SAMN03577196	SRS1260389
46	CFSAN031736	11	4	Enteritidis	5	2	3	7	6	6	11	SAL_EA9121AA	Livestock	Bovine	bovine minced meat	2005	Africa	Ethiopia			FDA Center for Food Safety and Applied Nutrition	PRJNA275961	SRP057873	SAMN03577269	SRS1260363
47	FDA00001501	11	4	Enteritidis	5	2	3	7	6	6	11	SAL_CA8258AA	Aquatic	Fish	frozen farm raised golden pompano whole round	2010	Asia	Taiwan			Food and Drug Administration - Center for Food Safety and Applied Nutrition (US)	PRJNA186035	SRP018785	SAMN02844886	SRS839253
48	99_CDC_H9556-sc-2013-02-27T10:27:04Z-150046	11	4	Enteritidis	5	2	3	7	6	6	11	SAL_BA9074AA									The Wellcome Trust Sanger Institute	PRJEB634	ERP001965	SAMEA2053457	ERS217246
49	UC12	11	4	Enteritidis	5	2	3	7	6	6	11	SAL_GA3001AA	Human	Human	Blood; homo sapiens	2002	North America	United States	Unresolved	Unresolved	The Genome Center at Washington University School of Medicine in St. Louis	PRJNA168789	SRP044976	SAMN03784387	SRS674603
50	0811210F	11	4	Enteritidis	5	2	3	7	6	6	11	SAL_GA3004AA	Wild Animal	Marine Mammal	abscess, liver; CA sea lion	2008	North America	United States	California		The Genome Center at Washington University School of Medicine in St. Louis	PRJNA168782	SRP044969	SAMN03784384	SRS674600
51	9810102B	11	4	Enteritidis	5	2	3	7	6	6	11	SAL_GA3024AA	Wild Animal	Marine Mammal	Abdomen; sea otter	1998	North America	United States	California		The Genome Center at Washington University School of Medicine in St. Louis	PRJNA168784	SRP044971	SAMN03784364	SRS674580
52	502571	11	4	Enteritidis	5	2	3	7	6	6	11	SAL_GA3006AA	Wild Animal	Marine Mammal	uterine; CA sea lion	2005	North America	United States	California		The Genome Center at Washington University School of Medicine in St. Louis	PRJNA168779	SRP044965	SAMN03784382	SRS674598
53	15207	11	4	Enteritidis	5	2	3	7	6	6	11	SAL_EA7418AA	Human	Human	Human; Homo sapiens		Europe	United Kingdom			Public Health England	PRJNA248792	SRP042645	SAMN04363650	SRS1219966
54	24057	11	4	Enteritidis	5	2	3	7	6	6	11	SAL_BA8745AA	Human	Human	Human; Homo sapiens	2014	Europe	United Kingdom	England		Public Health England - Gastrointestinal Bacteria Reference Unit	PRJNA248792	SRP042645	SAMN03479727	SRS906237
55	62557	11	4	Enteritidis	5	2	3	7	6	6	11	SAL_CA6746AA	Human	Human	Human; Homo sapiens	2014	Europe	United Kingdom		London	Gastrointestinal Bacteria Reference Unit, Public Health England	PRJNA248792	SRP042645	SAMN03465718	SRS897869
56	90475	11	4	Enteritidis	5	2	3	7	6	6	11	SAL_CA6895AA	Human	Human	Human; Homo sapiens	2015	Europe	United Kingdom			Public Health England - Gastrointestinal Bacteria Reference Unit	PRJNA248792	SRP042645	SAMN03460559	SRS893645
57	Enteritidis	11	4	Enteritidis	5	2	3	7	6	6	11	SAL_DA6792AA	Human	Human	clinical; human	2012	Europe	United Kingdom			Public Health England	PRJNA248792	SRP042645	SAMN02803727	SRS625572
58	2010K-0303	11	4	Enteritidis	5	2	3	7	6	6	11	SAL_GA3058AA	Human	Human	Stool; homo sapiens	2008	Asia	Thailand			The Genome Center at Washington University School of Medicine in St. Louis	PRJNA168734	SRP044803	SAMN02937557	SRS666146
59	15MN00098	11	4	Enteritidis	5	2	3	7	6	6	11	SAL_BA6397AA	Human	Human	Stool; Homo sapiens; Age: 50-59	2015	North America	United States	Minnesota		FOOD AND DRUG ADMINISTRATION, CENTER FOR FOOD SAFETY AND AP	PRJNA237212	SRP040026	SAMN03487590	SRS915009
60	2010K-0271	11	4	Enteritidis	5	2	3	7	6	6	11	SAL_GA3065AA	Human	Human	Blood; homo sapiens	2008	Asia	Thailand			The Genome Center at Washington University School of Medicine in St. Louis	PRJNA168727	SRP044795	SAMN02937439	SRS666070
61	54060	11	4	Enteritidis	5	2	3	7	6	6	11	SAL_CA6252AA	Human	Human	Human; Homo sapiens	2014	Europe	United Kingdom	England		Public Health England - Gastrointestinal Bacteria Reference Unit	PRJNA248792	SRP042645	SAMN03466218	SRS898375
62	9185	11	4	Enteritidis	5	2	3	7	6	6	11	SAL_BA9399AA	Human	Human	Human; Homo sapiens	2014	Europe	United Kingdom		London	Public Health England - Gastrointestinal Bacteria Reference Unit	PRJNA248792	SRP042645	SAMN03479415	SRS906015
63	PNUSAS000658	11	4	Enteritidis	5	2	3	7	6	6	11	SAL_EA1494AA	Human	Human	Stool; Age: 50-59	2015	North America	United States			Centers for Disease Control and Prevention - PulseNet	PRJNA230403	SRP040281	SAMN04017845	SRS1048156
64	25267	11	4	Enteritidis	5	2	3	7	6	6	11	SAL_CA4548AA	Human	Human	Human; Homo sapiens	2014	Europe	United Kingdom	England		Gastrointestinal Bacteria Reference Unit, Public Health England	PRJNA248792	SRP042645	SAMN03475494	SRS901962
65	MDH-2014-00937	11	4	Enteritidis	5	2	3	7	6	6	11	SAL_DA3820AA	Human	Human	feces; Homo sapiens	2014	North America	United States	Minnesota		Food and Drug Administration - Center for Food Safety and Applied Nutrition (US)	PRJNA237212	SRP040026	SAMN03013637	SRS693403
66	39509	11	4	Enteritidis	5	2	3	7	6	6	11	SAL_CA2325AA	Human	Human	Human; Homo sapiens	2014	Europe	United Kingdom		London	Gastrointestinal Bacteria Reference Unit, Public Health England	PRJNA248792	SRP042645	SAMN03477736	SRS904331
67	CFSAN024732	11	4	Enteritidis	5	2	3	7	6	6	11	SAL_EA4188AA	Poultry	Avian	Poultry egg	2012	South America	Chile	Arica y Parinacota Region	Arica	Food and Drug Administration - Center for Food Safety and Applied Nutrition (US)	PRJNA186035	SRP018785	SAMN03079677	SRS1041459
68	CFSAN023250	11	4	Enteritidis	5	2	3	7	6	6	11	SAL_DA3799AA	Human	Human	patient isolate; Homo sapiens	2006	North America	United States	Oregon		Food and Drug Administration - Center for Food Safety and Applied Nutrition (US)	PRJNA186035	SRP018785	SAMN02952836	SRS694852
69	92_3273-sc-2013-06-10T14:56:47Z-1611292	11	4	Enteritidis	5	2	3	7	6	6	11	SAL_BA0194AA				1992					The Wellcome Trust Sanger Institute	PRJEB634	ERP001965	SAMEA2080490	ERS255468
70	09:MF0001194R	11	4	Enteritidis	5	2	3	7	6	6	11	SAL_BA6372AA									The Wellcome Trust Sanger Institute	PRJEB2290	ERP000349	SAMEA1484295	ERS106261
71	P16-sc-2013-02-27T10:26:24Z-1500425	11	4	Enteritidis	5	2	3	7	6	6	11	SAL_BA9113AA				2007					The Wellcome Trust Sanger Institute	PRJEB634	ERP001965	SAMEA2053393	ERS217210
72	21122	11	4	Enteritidis	5	2	3	7	6	6	11	SAL_EA7867AA	Human	Human	Human; Homo sapiens		Europe	United Kingdom			Public Health England	PRJNA248792	SRP042645	SAMN04363135	SRS1219493
73	69283	11	4	Enteritidis	5	2	3	7	6	6	11	SAL_CA3379AA	Human	Human	Human; Homo sapiens	2014	Europe	United Kingdom	England		Gastrointestinal Bacteria Reference Unit, Public Health England	PRJNA248792	SRP042645	SAMN03476670	SRS903191
74	62600	11	4	Enteritidis	5	2	3	7	6	6	11	SAL_CA5982AA	Human	Human	Human; Homo sapiens	2014	Europe	United Kingdom			Public Health England - Gastrointestinal Bacteria Reference Unit	PRJNA248792	SRP042645	SAMN03466488	SRS898632
75	87348	11	4	Enteritidis	5	2	3	7	6	6	11	SAL_CA3380AA	Human	Human	Human; Homo sapiens	2015	Europe	United Kingdom	Wales		Gastrointestinal Bacteria Reference Unit, Public Health England	PRJNA248792	SRP042645	SAMN03476669	SRS903192
76	SA20083636	11	4	Enteritidis	5	2	3	7	6	6	11	SAL_DA9311AA	Environment	Soil/Dust	Environment - Litter/Manure	2008	North America	Canada	Ontario			PRJNA219482	SRP039437	SAMN02384211	SRS566059
77	38343	11	4	Enteritidis	5	2	3	7	6	6	11	SAL_CA2539AA	Human	Human	Human; Homo sapiens	2014	Europe	United Kingdom		London	Gastrointestinal Bacteria Reference Unit, Public Health England	PRJNA248792	SRP042645	SAMN03477522	SRS904061
78	38776	11	4	Enteritidis	5	2	3	7	6	6	11	SAL_BA9310AA	Human	Human	Human; Homo sapiens	2014	Europe	United Kingdom		London	Gastrointestinal Bacteria Reference Unit, Public Health England	PRJNA248792	SRP042645	SAMN03479504	SRS906006
79	FCC0035	11	4	Enteritidis	5	2	3	7	6	6	11	SAL_EA0593AA	Food	Plant	prima toast	2010	Asia	Philippines			FOOD AND DRUG ADMINISTRATION, CENTER FOR FOOD SAFETY AND AP	PRJNA186035	SRP018785	SAMN02344932	SRS509881
80	RD54-sc-2013-02-27T10:26:43Z-1500442	11	4	Enteritidis	5	2	3	7	6	6	11	SAL_BA9093AA				2005					The Wellcome Trust Sanger Institute	PRJEB634	ERP001965	SAMEA2053253	ERS217227
Reference	P125109 / NCTC 13349	11	4	Enteritidis	5	2	3	7	6	6	11	SAL_EA9783AA	Human	Human		1988					European Bioinformatics Institute	PRJEA30687		SAMEA1705941	ERS000072
81	MM_SW824_14_2	11	4	Enteritidis	5	2	3	7	6	6	11	SAL_BA4538AA									The Wellcome Trust Sanger Institute	PRJEB3186	ERP001671	SAMEA2020474	ERS159740
82	P542817	1981	4	Enteritidis	5	2	3	7	6	6	508	SAL_BA4534AA									The Wellcome Trust Sanger Institute	PRJEB3186	ERP001671	SAMEA2018882	ERS159742
83	LB_B20062_14_2	1981	4	Enteritidis	5	2	3	7	6	6	508	SAL_BA4569AA									The Wellcome Trust Sanger Institute	PRJEB3186	ERP001671	SAMEA2018876	ERS159724
84	LB_B20062_4	1981	4	Enteritidis	5	2	3	7	6	6	508	SAL_BA4581AA									The Wellcome Trust Sanger Institute	PRJEB3186	ERP001671	SAMEA2018775	ERS159718
85	80348	11	4	Enteritidis	5	2	3	7	6	6	11	SAL_CA4958AA	Human	Human	Human; Homo sapiens	2015	Europe	United Kingdom	England		Gastrointestinal Bacteria Reference Unit, Public Health England	PRJNA248792	SRP042645	SAMN03469912	SRS900855
86	CFSAN023244	11	4	Enteritidis	5	2	3	7	6	6	11	SAL_DA3805AA	Human	Human	patient isolate; Homo sapiens	2006	North America	United States	Oregon		FOOD AND DRUG ADMINISTRATION, CENTER FOR FOOD SAFETY AND AP	PRJNA186035	SRP018785	SAMN02952830	SRS694846
87	FMA0169	11	4	Enteritidis	5	2	3	7	6	6	11	SAL_DA6999AA	Environment	Plant	cumin seeds	2011	Asia	Syria			Food and Drug Administration - Center for Food Safety and Applied Nutrition (US)	PRJNA186035	SRP018785	SAMN02698233	SRS621672
88	39478	11	4	Enteritidis	5	2	3	7	6	6	11	SAL_CA4512AA	Human	Human	Human; Homo sapiens	2014	Europe	United Kingdom			Gastrointestinal Bacteria Reference Unit, Public Health England	PRJNA248792	SRP042645	SAMN03475530	SRS901987
89	73213	11	4	Enteritidis	5	2	3	7	6	6	11	SAL_CA4022AA	Human	Human	Human; Homo sapiens	2014	Europe	United Kingdom	England		Gastrointestinal Bacteria Reference Unit, Public Health England	PRJNA248792	SRP042645	SAMN03476027	SRS902582
90	73223	11	4	Enteritidis	5	2	3	7	6	6	11	SAL_CA2965AA	Human	Human	Human; Homo sapiens	2014	Europe	United Kingdom	England		Gastrointestinal Bacteria Reference Unit, Public Health England	PRJNA248792	SRP042645	SAMN03477084	SRS903621
91	H123760538	11	4	Enteritidis	5	2	3	7	6	6	11	SAL_DA0712AA	Human	Human	Human; Homo sapiens	2012	Europe	United Kingdom			Gastrointestinal Bacteria Reference Unit, Public Health England	PRJNA248792	SRP042645	SAMN03169273	SRS740612
92	68689	11	4	Enteritidis	5	2	3	7	6	6	11	SAL_CA0411AA	Human	Human	Human; Homo sapiens	2014	Europe	United Kingdom	England		Gastrointestinal Bacteria Reference Unit, Public Health England	PRJNA248792	SRP042645	SAMN03478878	SRS905398
93	A120	11	4	Enteritidis	5	2	3	7	6	6	11	SAL_BA4226AA									The Wellcome Trust Sanger Institute	PRJEB3215	ERP001718	SAMEA2057120	ERS223400
94	50364	11	4	Enteritidis	5	2	3	7	6	6	11	SAL_CA1384AA	Human	Human	Human; Homo sapiens	2014	Europe	United Kingdom	England		Gastrointestinal Bacteria Reference Unit, Public Health England	PRJNA248792	SRP042645	SAMN03478358	SRS904991
95	53113	11	4	Enteritidis	5	2	3	7	6	6	11	SAL_BA8372AA	Human	Human	Human; Homo sapiens	2014	Europe	United Kingdom	England		Public Health England - Gastrointestinal Bacteria Reference Unit	PRJNA248792	SRP042645	SAMN03479918	SRS906456
96	70695	1925	4	Enteritidis	454	2	3	7	6	6	11	SAL_CA4979AA	Human	Human	Human; Homo sapiens	2014	Europe	United Kingdom	England		Public Health England - Gastrointestinal Bacteria Reference Unit	PRJNA248792	SRP042645	SAMN03469888	SRS900834
97	H124860435	11	4	Enteritidis	5	2	3	7	6	6	11	SAL_DA0726AA	Human	Human	Human; Homo sapiens	2012	Europe	United Kingdom	England		Gastrointestinal Bacteria Reference Unit, Public Health England	PRJNA248792	SRP042645	SAMN03169246	SRS740598
98	10-05	11	4	Enteritidis	5	2	3	7	6	6	11	SAL_JA8487AA	Human	Human	Coproculture	2005	South America	Uruguay			Universidad de la Republica -Instituto de Higiene (Uruguay)	PRJEB2130		SAMEA811649	
99	H123300317	11	4	Enteritidis	5	2	3	7	6	6	11	SAL_DA0620AA	Human	Human	Human; Homo sapiens	2012	Europe	United Kingdom			Public Health England - Gastrointestinal Bacteria Reference Unit	PRJNA248792	SRP042645	SAMN03169407	SRS740725
100	H123900517	11	4	Enteritidis	5	2	3	7	6	6	11	SAL_DA0887AA	Human	Human	Human; Homo sapiens	2012	Europe	United Kingdom	England		Gastrointestinal Bacteria Reference Unit, Public Health England	PRJNA248792	SRP042645	SAMN03169043	SRS740428

Table S1: Metadata for the 203 *S.* Enteritidis genomes used in the phylogeny, including the reference.

					Achtman allele sequence type																				
number_ID	Name in EnteroBase	ST	eBG	Serotype (Predicted SISTR)	aroC	dnaN	hemD	hisD	purE	sucA	thrA	EnteroBase Uberstrain	Source Niche	Source Type	Source Details	Collection Year	Continent	Country	Region	City	Lab Contact	Bio Project ID	Project ID	Sample ID	Secondary Sample ID
101	53-94	11	4	Enteritidis	5	2	3	7	6	6	11	SAL_JA8481AA	Food	Meat	Pre-cooked chicken	1994	South America	Uruguay			Universidad de la Republica -Instituto de Higiene (Uruguay)	PRJEB2130		SAMEA811657	
102	206-99	11	4	Enteritidis	5	2	3	7	6	6	11	SAL_JA8484AA	Food	ND	Food	1999	South America	Uruguay			Universidad de la Republica -Instituto de Higiene (Uruguay)	PRJEB2130		SAMEA811656	
103	214-02	11	4	Enteritidis	5	2	3	7	6	6	11	SAL_JA8483AA	Human	Human	Haemoculture	2002	South America	Uruguay			Universidad de la Republica -Instituto de Higiene (Uruguay)	PRJEB2130		SAMEA811650	
104	EC20100325	11	4	Enteritidis	5	2	3	7	6	6	11	SAL_DA9201AA	Food	ND	ND	2010	North America	Canada	Ontario		PHAC	PRJNA219482	SRP039437	SAMN02384321	SRS566176
105	8-02	11	4	Enteritidis	5	2	3	7	6	6	11	SAL_JA8482AA	Human	Human	Coproculture	2002	South America	Uruguay			Universidad de la Republica -Instituto de Higiene (Uruguay)	PRJEB2130		SAMEA811652	
106	UY004-sc-2013-10-08T12:35:04Z-1711451	11	4	Enteritidis	5	2	3	7	6	6	11	SAL_AA9896AA				2001					The Wellcome Trust Sanger Institute	PRJEB4649	ERP003958	SAMEA2201433	ERS353810
107	251-01	11	4	Enteritidis	5	2	3	7	6	6	11	SAL_JA8488AA	Food	Avian	Eggs	2001	South America	Uruguay			Universidad de la Republica -Instituto de Higiene (Uruguay)	PRJEB2130		SAMEA811654	
108	37003	11	4	Enteritidis	5	2	3	7	6	6	11	SAL_CA3690AA	Human	Human	Human; Homo sapiens	2014	Europe	United Kingdom	England		Gastrointestinal Bacteria Reference Unit, Public Health England	PRJNA248792	SRP042645	SAMN03476360	SRS902861
109	65049	11	4	Enteritidis	5	2	3	7	6	6	11	SAL_CA3339AA	Human	Human	Human; Homo sapiens	2014	Europe	United Kingdom	England		Gastrointestinal Bacteria Reference Unit, Public Health England	PRJNA248792	SRP042645	SAMN03476710	SRS903342
110	NY-swgs1607	11	4	Enteritidis	5	2	3	7	6	6	11	SAL_BA1090AA	Human	Human	Urine; Homo sapiens	2013	North America	United States	New York	New York	NY State Dept. of Health, Wadsworth Center	PRJNA227458	SRP033012	SAMN03400128	SRS872148
111	NY56206572	11	4	Enteritidis	5	2	3	7	6	6	11	SAL_BA3146AA	Human	Human	Stool; Homo sapiens; Age: 20-29	2015	North America	United States	New York	New York	NY State Dept. of Health, Wadsworth Center	PRJNA227458	SRP033012	SAMN03776626	SRS961932
112	61979	11	4	Enteritidis	5	2	3	7	6	6	11	SAL_GA3030AA	Food	Meat	Ready to eat product; Pig	2002	North America	United States	Kansas		The Genome Center at Washington University School of Medicine in St. Louis	PRJNA168769	SRP044952	SAMN03784358	SRS674574
113	UC02	11	4	Enteritidis	5	2	3	7	6	6	11	SAL_GA3002AA	Human	Human	Stool; homo sapiens	2004	North America	United States	Unresolved	Unresolved	The Genome Center at Washington University School of Medicine in St. Louis	PRJNA168785	SRP044972	SAMN03784386	SRS674602
114	UC10	11	4	Enteritidis	5	2	3	7	6	6	11	SAL_GA3022AA	Human	Human	homo sapiens	2004	North America	United States	Unresolved	Unresolved	The Genome Center at Washington University School of Medicine in St. Louis	PRJNA168787	SRP044974	SAMN03784366	SRS674582
115	UC06	11	4	Enteritidis	5	2	3	7	6	6	11	SAL_GA2992AA	Human	Human	Blood; homo sapiens	2004					The Genome Center at Washington University School of Medicine in St. Louis	PRJNA168940	SRP044988	SAMN03784396	SRS674612
116	NY-swgs1281	11	4	Enteritidis	5	2	3	7	6	6	11	SAL_DA6740AA	Human	Human	Blood; Homo sapiens	2014	North America	United States	New York	New York	NY State Dept. of Health, Wadsworth Center	PRJNA227458	SRP033012	SAMN02797523	SRS627301
117	WAPHL_SAL-A01147	11	4	Enteritidis	5	2	3	7	6	6	11	SAL_EA8164AA	Human	Human	Homo sapiens; Stool	2015	North America	United States	Washington		FDA Center for Food Safety and Applied Nutrition	PRJNA252015	SRP047173	SAMN04358656	SRS1221881
118	UC11	11	4	Enteritidis	5	2	3	7	6	6	11	SAL_GA3021AA	Human	Human	Blood; homo sapiens	2001	North America	United States	Unresolved	Unresolved	The Genome Center at Washington University School of Medicine in St. Louis	PRJNA168788	SRP044975	SAMN03784367	SRS674583
119	EC20120200	11	4	Enteritidis	5	2	3	7	6	6	11	SAL_DA9272AA	Food	ND	ND	2010	North America	Canada	Nova Scotia		Public Health Agency Canada	PRJNA219482	SRP039437	SAMN02384250	SRS566098
120	swgs1203	11	4	Enteritidis	5	2	3	7	6	6	11	SAL_DA8947AA	Human	Human	Stool; Homo sapiens	2014	North America	United States	New York	New York	FSIS SRCAMB	PRJNA227458	SRP033012	SAMN02676369	SRS568932
121	UC13	11	4	Enteritidis	5	2	3	7	6	6	11	SAL_GA3020AA	Human	Human	Blood; homo sapiens	2009	North America	United States	Unresolved	Unresolved	The Genome Center at Washington University School of Medicine in St. Louis	PRJNA168790	SRP044977	SAMN03784368	SRS674584
122	2702-sc-2012-11-09T12:21:21Z-1500164	11	4	Enteritidis	5	2	3	7	6	6	11	SAL_BA5678AA				1996					The Wellcome Trust Sanger Institute	PRJEB634	ERP001965	SAMEA1712248	ERS188228
123	21065	11	4	Enteritidis	5	2	3	7	6	6	11	SAL_EA7045AA	Human	Human	Human; Homo sapiens	2014	Europe	United Kingdom	England		Public Health England	PRJNA248792	SRP042645	SAMN04364029	SRS1220382
124	8-89	1974	4	Enteritidis	5	2	378	7	6	6	11	SAL_JA8485AA	Human	Human	Haemoculture	1989	South America	Uruguay			Universidad de la Republica -Instituto de Higiene (Uruguay)	PRJEB2130		SAMEA811659	
125	31-88	1974	4	Enteritidis	5	2	378	7	6	6	11	SAL_JA8486AA	Human	Human	Coproculture	1988	South America	Uruguay			Universidad de la Republica -Instituto de Higiene (Uruguay)	PRJEB2130		SAMEA811661	
126	PNUSAS001296	1974	4	Enteritidis	5	2	378	7	6	6	11	SAL_EA6307AA	Human	Human	Urine; Age: 0-4	2014	North America	United States			Centers for Disease Control and Prevention Enteric Diseases Laboratory Branch	PRJNA230403	SRP040281	SAMN04330126	SRS1207290
127	PNUSAS001293	1974	4	Enteritidis	5	2	378	7	6	6	11	SAL_EA6310AA	Human	Human	Stool; Age: 40-49	2014	North America	United States			Centers for Disease Control and Prevention Enteric Diseases Laboratory Branch	PRJNA230403	SRP040281	SAMN04330123	SRS1207271
128	PNUSAS001292	1974	4	Enteritidis	5	2	378	7	6	6	11	SAL_EA6311AA				2013	North America	United States			Centers for Disease Control and Prevention Enteric Diseases Laboratory Branch	PRJNA230403	SRP040281	SAMN04330122	SRS1207272
129	351865	1974	4	Enteritidis	5	2	378	7	6	6	11	SAL_GA3068AA				2009					The Genome Center at Washington University School of Medicine in St. Louis	PRJNA168724	SRP044792	SAMN02937423	SRS666066
130	39508	11	4	Enteritidis	5	2	3	7	6	6	11	SAL_CA1179AA	Human	Human	Human; Homo sapiens	2014	Europe	United Kingdom	England		Gastrointestinal Bacteria Reference Unit, Public Health England	PRJNA248792	SRP042645	SAMN03478497	SRS905129
131	7_55-sc-2013-06-10T14:58:01Z-1611344	11	4	Enteritidis	5	2	3	7	6	6	11	SAL_BA0158AA				1955					The Wellcome Trust Sanger Institute	PRJEB634	ERP001965	SAMEA2078104	ERS255520
132	CFSAN034131	11	4	Enteritidis	5	2	3	7	6	6	11	SAL_BA2513AA	Poultry	Avian	product-eggs-raw-yolks	2012	North America	United States	Nebraska		FOOD AND DRUG ADMINISTRATION, CENTER FOR FOOD SAFETY AND AP	PRJNA242847	SRP040648	SAMN03763520	SRS972592
133	CFSAN031777	11	4	Enteritidis	5	2	3	7	6	6	11	SAL_EA8245AA	Human	Human	feces; Homo sapiens	2013	Africa	Ethiopia			FDA Center for Food Safety and Applied Nutrition	PRJNA275961	SRP057873	SAMN03577310	SRS1232487
134	83682	11	4	Enteritidis	5	2	3	7	6	6	11	SAL_CA2411AA	Human	Human	Human; Homo sapiens	2014	Europe	United Kingdom	England		Public Health England - Gastrointestinal Bacteria Reference Unit	PRJNA248792	SRP042645	SAMN03477650	SRS904271
135	H124000530	11	4	Enteritidis	5	2	3	7	6	6	11	SAL_DA0516AA	Human	Human	Human; Homo sapiens	2012	Europe	United Kingdom	England		Gastrointestinal Bacteria Reference Unit, Public Health England	PRJNA248792	SRP042645	SAMN03169547	SRS740842
136	H123640722	11	4	Enteritidis	5	2	3	7	6	6	11	SAL_DA0597AA	Human	Human	Human; Homo sapiens	2012					Gastrointestinal Bacteria Reference Unit, Public Health England	PRJNA248792	SRP042645	SAMN03169448	SRS740748
137	H122580622	11	4	Enteritidis	5	2	3	7	6	6	11	SAL_DA0696AA	Human	Human	Human; Homo sapiens	2012	Europe	United Kingdom			Gastrointestinal Bacteria Reference Unit, Public Health England	PRJNA248792	SRP042645	SAMN03169294	SRS740627
138	63933	11	4	Enteritidis	5	2	3	7	6	6	11	SAL_BA8293AA	Human	Human	Human; Homo sapiens	2014	Europe	United Kingdom	England		Public Health England - Gastrointestinal Bacteria Reference Unit	PRJNA248792	SRP042645	SAMN03479958	SRS906493
139	H124380523	11	4	Enteritidis	5	2	3	7	6	6	11	SAL_DA0436AA	Human	Human	Human; Homo sapiens	2012					Gastrointestinal Bacteria Reference Unit, Public Health England	PRJNA248792	SRP042645	SAMN03169632	SRS740940
140	21132	11	4	Enteritidis	5	2	3	7	6	6	11	SAL_EA7763AA	Human	Human	Human; Homo sapiens	2014	Europe	United Kingdom	England		Public Health England	PRJNA248792	SRP042645	SAMN04363239	SRS1219604
141	78697	11	4	Enteritidis	5	2	3	7	6	6	11	SAL_CA4983AA	Human	Human	Human; Homo sapiens	2014	Europe	United Kingdom	England		Gastrointestinal Bacteria Reference Unit, Public Health England	PRJNA248792	SRP042645	SAMN03469884	SRS900833
142	49742	11	4	Enteritidis	5	2	3	7	6	6	11	SAL_CA6060AA	Human	Human	Human; Homo sapiens	2014	Europe	United Kingdom	England		Public Health England - Gastrointestinal Bacteria Reference Unit	PRJNA248792	SRP042645	SAMN03466410	SRS898565
143	45619	11	4	Enteritidis	5	2	3	7	6	6	11	SAL_CA2997AA	Human	Human	Human; Homo sapiens	2014	Europe	United Kingdom		London	Gastrointestinal Bacteria Reference Unit, Public Health England	PRJNA248792	SRP042645	SAMN03477052	SRS903605
144	66884	11	4	Enteritidis	5	2	3	7	6	6	11	SAL_CA5287AA	Human	Human	Human; Homo sapiens	2014	Europe	United Kingdom			Public Health England - Gastrointestinal Bacteria Reference Unit	PRJNA248792	SRP042645	SAMN03469158	SRS900483
145	H122860674	11	4	Enteritidis	5	2	3	7	6	6	11	SAL_DA1117AA	Human	Human	Human; Homo sapiens	2012	Europe	United Kingdom		London	Gastrointestinal Bacteria Reference Unit, Public Health England	PRJNA248792	SRP042645	SAMN03168752	SRS740048
146	6497	11	4	Enteritidis	5	2	3	7	6	6	11	SAL_EA7432AA	Human	Human	Human; Homo sapiens		Europe	United Kingdom			Public Health England	PRJNA248792	SRP042645	SAMN04363636	SRS1219949
147	PNUSAS000287	11	4	Enteritidis	5	2	3	7	6	6	11	SAL_BA5119AA	Human	Human	Stool; Age: 30-39	2015	North America	United States			Centers for Disease Control and Prevention - PulseNet	PRJNA230403	SRP040281	SAMN03579619	SRS932284
148	MDH-2014-00496	11	4	Enteritidis	5	2	3	7	6	6	11	SAL_DA3222AA	Wild Animal	Reptile	Bearded Dragon Feces; Pogona vitticeps	2003	North America	United States	Minnesota		Food and Drug Administration - Center for Food Safety and Applied Nutrition (US)	PRJNA215333	SRP032981	SAMN02699662	SRS700465
149	PNUSAS000478	11	4	Enteritidis	5	2	3	7	6	6	11	SAL_EA1628AA				2015	North America	United States			EDLB-CDC	PRJNA230403	SRP040281	SAMN03793015	SRS1047124
150	FSE0158	11	4	Enteritidis	5	2	3	7	6	6	11	SAL_DA6158AA	Food	Plant	pepper, dried	2011	Africa	Ethiopia			Food and Drug Administration - Center for Food Safety and Applied Nutrition (US)	PRJNA186035	SRP018785	SAMN02698426	SRS643706
151	EC20120051	11	4	Enteritidis	5	2	3	7	6	6	11	SAL_DA9200AA	Livestock	Bovine	ANIMAL-Domestic Cattle	2011	North America	Canada	Ontario		PHAC	PRJNA219482	SRP039437	SAMN02384322	SRS566177
152	EC20121812	11	4	Enteritidis	5	2	3	7	6	6	11	SAL_DA9230AA	Food	ND	ND	2011	North America	Canada	British Columbia		PHAC	PRJNA219482	SRP039437	SAMN02384292	SRS566140
153	CFSAN028609	11	4	Enteritidis	5	2	3	7	6	6	11	SAL_CA7909AA	Poultry	Avian	chicken	2014	North America	United States	Minnesota		FOOD AND DRUG ADMINISTRATION, CENTER FOR FOOD SAFETY AND AP	PRJNA186035	SRP018785	SAMN03282117	SRS850598
154	2014K-0501-2	11	4	Enteritidis	5	2	3	7	6	6	11	SAL_CA9408AA					North America	United States			Enteric Diseases Laboratory Branch, Centers for Disease Control and Prevention	PRJNA230403	SRP040281	SAMN03253248	SRS786053
155	NY58703765	11	4	Enteritidis	5	2	3	7	6	6	11	SAL_EA4107AA	Human	Human	Blood; Homo sapiens	2015	North America	United States	New York	New York	NY State Dept. of Health, Wadsworth Center	PRJNA227458	SRP033012	SAMN04044037	SRS1061529
156	AZ_TG71071	11	4	Enteritidis	5	2	3	7	6	6	11	SAL_DA9077AA	Poultry	Avian	Chicken breast tenderloins	2013	North America	United States	Florida			PRJNA183847	SRP028894	SAMN02595014	SRS568046
157	swgs1053	11	4	Enteritidis	5	2	3	7	6	6	11	SAL_DA8923AA	Human	Human	Blood; Homo sapiens	2013	North America	United States	New York	New York	NY State Dept. of Health, Wadsworth Center	PRJNA227458	SRP033012	SAMN02676317	SRS569067
158	2014K-0526	11	4	Enteritidis	5	2	3	7	6	6	11	SAL_DA2042AA	Human	Human	Stool; Age: 20-29	2014	North America	United States			Enteric Diseases Laboratory Branch, Centers for Disease Control and Prevention	PRJNA230403	SRP040281	SAMN03098815	SRS724542
159	FCC0050	11	4	Enteritidis	5	2	3	7	6	6	11	SAL_EA0494AA	Environment	Soil/Dust	swab	2010	North America	United States	Indiana		Food and Drug Administration - Center for Food Safety and Applied Nutrition (US)	PRJNA186035	SRP018785	SAMN02344870	SRS514202
160	PNUSAS000145	11	4	Enteritidis	5	2	3	7	6	6	11	SAL_CA8313AA				2014	North America	United States			Centers for Disease Control and Prevention - PulseNet	PRJNA230403	SRP040281	SAMN03295014	SRS834205
161	NY-30658	11	4	Enteritidis	5	2	3	7	6	6	11	SAL_CA9436AA	Poultry	Avian	Chicken Breast	2003	North America	United States	Maryland		Food and Drug Administration - Center for Food Safety and Applied Nutrition (US)	PRJNA183850	SRP028607	SAMN02777709	SRS783188
162	15MN00345	11	4	Enteritidis	5	2	3	7	6	6	11	SAL_BA1339AA	Human	Human	feces; Homo sapiens	2015	North America	United States	Minnesota		Food and Drug Administration - Center for Food Safety and Applied Nutrition (US)	PRJNA237212	SRP040026	SAMN03946227	SRS1021274
163	PNUSAS000233	11	4	Enteritidis	5	2	3	7	6	6	11	SAL_BA5156AA	Human	Human	Stool; Age: 0-4	2015	North America	United States			Centers for Disease Control and Prevention	PRJNA230403	SRP040281	SAMN03567175	SRS932271
164	MOD1-Lipp-402	11	4	Enteritidis	5	2	3	7	6	6	11	SAL_EA5553AA	Environment	Water	Little River; water	2006	North America	United States	Georgia		Food and Drug Administration - Center for Food Safety and Applied Nutrition (US)	PRJNA186035	SRP018785	SAMN04296489	SRS1183876
165	EC20130348	11	4	Enteritidis	5	2	3	7	6	6	11	SAL_DA9210AA	Poultry	Avian	Chicken	2010	North America	Canada	Ontario		PHAC	PRJNA219482	SRP039437	SAMN02384312	SRS566167
166	CFSAN018518	11	4	Enteritidis	5	2	3	7	6	6	11	SAL_DA6032AA	Aquatic	Marine Mammal	Sea lion (rectal - swab)	2007	North America	United States	Alaska		Food and Drug Administration - Center for Food Safety and Applied Nutrition (US)	PRJNA186035	SRP018785	SAMN02849786	SRS650459
167	93569	11	4	Enteritidis	5	2	3	7	6	6	11	SAL_CA3376AA	Human	Human	Human; Homo sapiens	2015	Europe	United Kingdom			Gastrointestinal Bacteria Reference Unit, Public Health England	PRJNA248792	SRP042645	SAMN03476673	SRS903188
168	15211	11	4	Enteritidis	5	2	3	7	6	6	11	SAL_EA7711AA	Human	Human	Human; Homo sapiens		Europe	United Kingdom			Public Health England	PRJNA248792	SRP042645	SAMN04363292	SRS1219660
169	CDC_2010K-0968	11	4	Enteritidis	5	2	3	7	6	6	11	SAL_CA1336AA	Human	Human	Clinical; Homo sapiens	2010	North America	United States	Ohio		FOOD AND DRUG ADMINISTRATION, CENTER FOR FOOD SAFETY AND AP	PRJNA52491	SRP014062	SAMN01041170	SRS348552
170	CFSAN030818	11	4	Enteritidis	5	2	3	7	6	6	11	SAL_BA5700AA	Food	Avian	product-eggs-raw-yolks	2012	North America	United States	Indiana		Food and Drug Administration - Center for Food Safety and Applied Nutrition (US)	PRJNA186035	SRP018785	SAMN03458410	SRS922822
171	PNUSAS000700	11	4	Enteritidis	5	2	3	7	6	6	11	SAL_EA1501AA	Human	Human	Age: 50-59; Stool	2015	North America	United States			Centers for Disease Control and Prevention - PulseNet	PRJNA230403	SRP040281	SAMN04001738	SRS1048032
172	85366	11	4	Enteritidis	5	2	3	7	6	6	11	SAL_GA3032AA	Poultry	Avian	Ground chicken; Chicken	2007	North America	United States			The Genome Center at Washington University School of Medicine in St. Louis	PRJNA168767	SRP044949	SAMN03784356	SRS674572
173	2009K-0958	11	4	Enteritidis	5	2	3	7	6	6	11	SAL_GA3070AA	Human	Human	homo sapiens	2009	North America	United States			The Genome Center at Washington University School of Medicine in St. Louis	PRJNA168722	SRP044712	SAMN02937421	SRS666064
174	NY-swgs1585	11	4	Enteritidis	5	2	3	7	6	6	11	SAL_BA1114AA	Human	Human	Other; Homo sapiens	2014	North America	United States	New York	New York	NY State Dept. of Health, Wadsworth Center	PRJNA227458	SRP033012	SAMN03316883	SRS829611
175	swgs1030	11	4	Enteritidis	5	2	3	7	6	6	11	SAL_DA8938AA	Human	Human	Stool; Homo sapiens	2013	North America	United States	New York	New York	NY State Dept. of Health, Wadsworth Center	PRJNA227458	SRP033012	SAMN02676294	SRS569049
176	NY-swgs1590	11	4	Enteritidis	5	2	3	7	6	6	11	SAL_BA1109AA	Human	Human	Stool; Homo sapiens	2015	North America	United States	New York	New York	NY State Dept. of Health, Wadsworth Center	PRJNA227458	SRP033012	SAMN03339155	SRS841921
177	VA-WGS-00657	11	4	Enteritidis	5	2	3	7	6	6	11	SAL_EA8492AA	Environment	Water	ND	2014	North America	United States	Virginia		FDA Center for Food Safety and Applied Nutrition	PRJNA219491	SRP033708	SAMN04362110	SRS1235900
178	SA20090877	11	4	Enteritidis	5	2	3	7	6	6	11	SAL_DA9280AA	Food	ND	ND	2009	North America	Canada	Saskatchewan		Public Health Agency Canada	PRJNA219482	SRP039437	SAMN02384242	SRS566090
179	SA20093784	11	4	Enteritidis	5	2	3	7	6	6	11	SAL_DA9292AA	Food	ND	ND	2009	North America	Canada	British Columbia			PRJNA219482	SRP039437	SAMN02384230	SRS566078
180	SA20093977	11	4	Enteritidis	5	2	3	7	6	6	11	SAL_DA9291AA	Poultry	Avian	Chicken	2009	North America	Canada	British Columbia			PRJNA219482	SRP039437	SAMN02384231	SRS566079
181	S-380	11	4	Enteritidis	5	2	3	7	6	6	11	SAL_GA3035AA	Food	Plant	Foam	2008					The Genome Center at Washington University School of Medicine in St. Louis	PRJNA168763	SRP044928	SAMN03784353	SRS674569
182	EC20120590	11	4	Enteritidis	5	2	3	7	6	6	11	SAL_DA9196AA	Poultry	Avian	Chicken	2011	North America	Canada	Nova Scotia			PRJNA219482	SRP039437	SAMN02384326	SRS566181
183	15MN00139	11	4	Enteritidis	5	2	3	7	6	6	11	SAL_BA5669AA	Human	Human	Stool; Homo sapiens; Age: 70-79	2015	North America	United States	Minnesota		FOOD AND DRUG ADMINISTRATION, CENTER FOR FOOD SAFETY AND AP	PRJNA237212	SRP040026	SAMN03492572	SRS923001
184	CFSAN034774	11	4	Enteritidis	5	2	3	7	6	6	11	SAL_EA4868AA	Human	Human	human isolate; Homo sapiens	2014	North America	United States	Washington		CFSAN	PRJNA186035	SRP018785	SAMN03787673	SRS1154417
185	AZ_TG71151	11	4	Enteritidis	5	2	3	7	6	6	11	SAL_DA8696AA	Poultry	Avian	Boneless skinless chicken breast	2013	North America	United States	Alabama		Food and Drug Administration - Center for Food Safety and Applied Nutrition (US)	PRJNA183847	SRP028894	SAMN02595034	SRS580075
186	NY-swgs1622	11	4	Enteritidis	5	2	3	7	6	6	11	SAL_BA1076AA	Human	Human	Stool; Homo sapiens	2015	North America	United States	New York	New York	NY State Dept. of Health, Wadsworth Center	PRJNA227458	SRP033012	SAMN03421027	SRS875138
187	PNUSAS000079	11	4	Enteritidis	5	2	3	7	6	6	11	SAL_CA9818AA	Human	Human	Stool; Age: 20-29	2014	North America	United States			Enteric Diseases Laboratory Branch, Centers for Disease Control and Prevention	PRJNA230403	SRP040281	SAMN03218529	SRS753524
188	FSIS1504441	11	4	Enteritidis	5	2	3	7	6	6	11	SAL_EA2245AA	Poultry	Avian	Comminuted Chicken	2015	North America	United States	Illinois		Department of Agriculture - Food Safety and Inspection Service (US)	PRJNA242847	SRP040648	SAMN04101286	SRS1080105
189	SA20090435	11	4	Enteritidis	5	2	3	7	6	6	11	SAL_DA9288AA	Poultry	Avian	Chicken	2008	North America	Canada	British Columbia		Public Health Agency Canada	PRJNA219482	SRP039437	SAMN02384234	SRS566082
190	PNUSAS001383	11	4	Enteritidis	5	2	3	7	6	6	11	SAL_EA8873AA	Human	Human	Blood; Age: 70-79	2015	North America	United States			Centers for Disease Control and Prevention Enteric Diseases Laboratory Branch	PRJNA230403	SRP040281	SAMN04412499	SRS1249569
191	CVM-N15781	11	4	Enteritidis	5	2	3	7	6	6	11	SAL_EA2573AA	Poultry	Avian	chicken breast	2007	North America	United States	New Mexico		Food and Drug Administration (US)	PRJNA271470	SRP061987	SAMN03842316	SRS1092896
192	IEH-NGS-SAL-02458	11	4	Enteritidis	5	2	3	7	6	6	11	SAL_BA7279AA	Poultry	Avian	chicken	2014	North America	United States	Colorado		FOOD AND DRUG ADMINISTRATION, CENTER FOR FOOD SAFETY AND AP	PRJNA186035	SRP018785	SAMN03455247	SRS909462
193	15MN00255	11	4	Enteritidis	5	2	3	7	6	6	11	SAL_BA1897AA	Poultry	Composite Food	frozen raw stuffed chicken product	2015	North America	United States	Minnesota		FOOD AND DRUG ADMINISTRATION, CENTER FOR FOOD SAFETY AND AP	PRJNA215333	SRP032981	SAMN03854519	SRS992395
194	PNUSAS000108	2704	4	Enteritidis	5	2	360	7	6	6	589	SAL_CA9649AA	Human	Human	Stool; Age: 20-29	2014	North America	United States			Centers for Disease Control and Prevention - PulseNet	PRJNA230403	SRP040281	SAMN03246957	SRS779826
195	CFSAN023319	11	4	Enteritidis	5	2	3	7	6	6	11	SAL_DA3430AA	Human	Human	feces; Homo sapiens	2011	North America	United States	Minnesota		Food and Drug Administration - Center for Food Safety and Applied Nutrition (US)	PRJNA183850	SRP028607	SAMN02989035	SRS697277
196	AZ-TG74480	11	4	Enteritidis	5	2	3	7	6	6	11	SAL_DA1938AA	Poultry	Avian	Chicken Breast	2005	North America	United States	Maryland		Food and Drug Administration - Center for Food Safety and Applied Nutrition (US)	PRJNA183847	SRP028894	SAMN02894019	SRS731495
197	EC20120356	11	4	Enteritidis	5	2	3	7	6	6	11	SAL_DA9199AA	Livestock	Bovine	ANIMAL-Domestic Cattle	2011	North America	Canada	Ontario		PHAC	PRJNA219482	SRP039437	SAMN02384323	SRS566178
198	MDH-2014-00890	11	4	Enteritidis	5	2	3	7	6	6	11	SAL_DA5246AA	Human	Human	urine; Homo sapiens	2014	North America	United States	Minnesota		Food and Drug Administration - Center for Food Safety and Applied Nutrition (US)	PRJNA237212	SRP040026	SAMN02947311	SRS673889
199	ASM_13	11	4	Enteritidis	5	2	3	7	6	6	11	SAL_EA1766AA	Environment	Soil/Dust	environmental swab	2015	North America	United States			FDA	PRJNA295366	SRP063605	SAMN04046592	SRS1064796
200	CFSAN035309	11	4	Enteritidis	5	2	3	7	6	6	11	SAL_BA1041AA	Food	Avian	product-eggs-raw-yolks	2012	North America	United States	Iowa		Food and Drug Administration - Center for Food Safety and Applied Nutrition (US)	PRJNA242847	SRP040648	SAMN03838271	SRS1034421
201	CFSAN032351	11	4	Enteritidis	5	2	3	7	6	6	11	SAL_BA4154AA	Poultry	Avian	chicken	2014	North America	United States	Nebraska		Food and Drug Administration - Center for Food Safety and Applied Nutrition (US)	PRJNA186035	SRP018785	SAMN03487677	SRS947969
202	SA20094383	11	4	Enteritidis	5	2	3	7	6	6	11	SAL_DA9285AA	Food	ND	ND	2009	North America	Canada	Ontario		PHAC	PRJNA219482	SRP039437	SAMN02384237	SRS566085

(*) Annotated as “before 2008”

Table S2: Summary results of the PHAST server prophage identification.

Strain name	NCBI accession	Length*	PHAST Completeness	PHAST Score	CDS †	Prophage position (start-stop) in the sequence	GC (%)
31/88 and 8/89	-	44Kb	intact	150	60	1-44067	50.11
SL1344	FQ312003.1	67Kb	intact	150	68	2713417-2780416	51.26
DT104	HF937208.1	45Kb	intact	140	66	1954176-1999250	50.85
USDA-ARS-USMARC-1880	CP014981.1	45.6 Kb	intact	150	61	1307822-1353479	49.99

*-Length of the identified prophage region.
†- Number of coding sequences (CDS) contained in the region.

The PHAST analyses were performed using the RASTtk annotation of each sequence. For the complete genomes of *S.* Typhimurium strains SL1344, DT104 and USDA-ARS-USMARC-1880, here we only show the identification parameters obtained for those prophages that shared nucleotide identity with the 44 kbp contigs sequences of strains 31/88 and 8/89 (see Fig. 1).

Table S3: Annotation of the 44 kbp contig by RASTtk.
For each CDS the RASTtk annotation is shown along with the identification obtained by PHAST. For each annotated CDS, the percent of nucleotide identity and coverage respect to the genes within the prophage regions identified by PHAST in S. Typhimurium SL1344 and DT104 strains (see Figure 1) is also shown.

							**SL1344**		**DT104**	
**feature_id**	**type**	**start**	**stop**	**strand**	**RASTtk identification**	**PHAST identification**	**% nucleotide identity**	**% coverage**	**% nucleotide identity**	**% coverage**
1	CDS	3	134	+	Gifsy-1 prophage (*)	virulence protein GogA (*)	93	100		
2	CDS	1065	268	-	Putative prophage antitermination protein	putative antitermination protein				
3	CDS	1201	1055	-	Phage protein	conserved hypothetical bacteriophage protein	94	100		
4	CDS	1809	1198	-	Phage protein	NinG	100	100		
5	CDS	2012	1812	-	FIG01045325: hypothetical protein	hypothetical protein STM2620.1n.Gifsy1	100	100		
6	CDS	2620	2018	-	Gifsy-2 prophage protein STM1020/STM2620	conserved hypothetical bacteriophage protein	100	100		
7	CDS	2771	2655	-	Phage protein	hypothetical protein	100	100		
8	CDS	3253	3020	-	DNA-damage-inducible protein I	bacteriophage putative DNA damage-inducible protein	100	100		
9	CDS	3697	3512	-	hypothetical protein	hypothetical protein	100	100		
10	CDS	4096	3815	-	Phage protein (ACLAME 31)	hypothetical protein PhiV10p54	100	100		
11	CDS	4742	4089	-	Phage EaA protein	EaA	100	100		
12	CDS	5215	4745	-	SAM-dependent methyltransferase	putative methylase	100	100		
13	CDS	5882	5217	-	Phage EaD protein	EaD-like protein	100	100		
14	CDS	6589	5897	-	Phage DNA replication protein P	replication protein 14	100	100		
15	CDS	7491	6586	-	Replication protein O	replication protein O	100	100		
16	CDS	7903	7583	-	Gifsy-1 prophage cI	probable regulatory protein	100	100		
17	CDS	8150	7923	-	hypothetical protein	conserved hypothetical prophage protein	100	100		
18	CDS	8164	8631	+	hypothetical protein	prophage repressor	100	100		
19	CDS	8783	9868	+	ATPase domain protein	putative ParA protein	100	100		
20	CDS	10182	9976	-	FIG01046771: hypothetical protein	hypothetical protein	100	100		
21	CDS	10473	10589	+	Phage protein	hypothetical protein STM2630.1n.Gifsy1	100	100		
22	CDS	10582	10752	+	FIG01045596: hypothetical protein	hypothetical protein STM2630.Gifsy1	100	100		
23	CDS	10893	14093	+	Gifsy-1 prophage RecE	bacteriophage exodeoxyribonuclease VIII-like protein	95	100	97	95
24	CDS	14104	15213	+	Gifsy-2 prophage RecT	RecT	97	100		
25	CDS	15256	15495	+	FIG00643583: hypothetical protein	hypothetical protein STM2634.Gifsy1	91	100		
26	CDS	17027	15798	-	Phage integrase family protein	bacteriophage integrase protein	98	100		
27	CDS	17678	19168	+	**leucine-rich repeat protein (GogB)**	leucine-rich repeat protein	99	99		
28	CDS	19381	19569	+	Mobile element protein	putative transposase	98	100		
29	CDS	20148	19930	-	Error-prone, lesion bypass DNA polymerase V (UmuC)	error-prone lesion bypass DNA polymerase V				
30	CDS	20401	21408	+	**putative cytoplasmic protein (SseK3)**	hypothetical protein sb26				
31	CDS	21413	21547	+	hypothetical protein	-				
32	CDS	22261	21692	-	phage tail assembly-like protein	tail fibers protein	83	99	87	99
33	CDS	23712	22261	-	Putative tail fiber protein	bacteriophage side tail fiber protein	90	63	93	99
34	CDS	24304	23702	-	IS, phage, Tn; Transposon-related functions	hypothetical protein			99	100
35	CDS	25547	24306	-	IS, phage, Tn; Transposon-related functions	putative baseplate J-like protein			99	100
36	CDS	25900	25544	-	FIG00465618: hypothetical protein	hypothetical protein			100	100
37	CDS	26590	25913	-	phage P2 baseplate assembly protein gpV	putative baseplate protein			99	100
38	CDS	27440	26571	-	FIG00641787: hypothetical protein	hypothetical protein			100	100
39	CDS	27739	27437	-	FIG00925000: hypothetical protein	putative structural protein			100	100
40	CDS	28449	27739	-	FIG01047071: hypothetical protein	hypothetical protein			98	100
41	CDS	30617	28446	-	Membrane-bound lytic murein transglycosylase D	tail tape measure protein			99	100
42	CDS	30780	30601	-	hypothetical protein	-			100	100
43	CDS	31229	30825	-	FIG00460725: hypothetical protein	hypothetical protein			100	100
44	CDS	31675	31229	-	FIG00466839: hypothetical protein	hypothetical protein			99	100
45	CDS	33160	31676	-	Phage protein	hypothetical protein			100	100
46	CDS	33686	33141	-	FIG00458318: hypothetical protein	hypothetical protein			100	100
47	CDS	34036	33671	-	Phage protein	hypothetical protein			99	100
48	CDS	34617	34033	-	Phage protein	hypothetical protein			99	100
49	CDS	35057	34611	-	FIG01049552: hypothetical protein	hypothetical protein OP2_ORF6			97	100
50	CDS	35411	35064	-	COG1132: ABC-type multidrug transport system, ATPase and permease components	hypothetical protein			99	100
51	CDS	36443	35415	-	FIG00468159: hypothetical protein	major capsid protein			100	100
52	CDS	36925	36443	-	FIG01049577: hypothetical protein	hypothetical protein			99	100
53	CDS	38273	36927	-	Phage protein	prohead protease			99	100
54	CDS	38959	38270	-	Plasmid-related protein	putative head protein			99	100
55	CDS	40520	39000	-	Phage protein	putative portal protein			98	100
56	CDS	42139	40520	-	Terminase large subunit	terminase large subunit			92	4
57	CDS	42768	42142	-	FIG01045330: hypothetical protein	terminase small subunit			94	11
58	CDS	43079	42873	-	hypothetical protein	hypothetical protein				
59	CDS	43369	43187	-	FIG01047729: hypothetical protein	hypothetical protein			100	100
60	CDS	44067	43594	-	Rz endopeptidase from lambdoid phage	bacteriophage lysis protein	100	100	91	100

**Color key**	**Note**
regulatory or replication phage proteins	(*) Partial sequence, which is completed when the prophage is in the genome context.
structural phage proteis	
moron genes	

Table S4: Metadata corresponding to 154 ST-1974 *S.* Enteritidis genomes found in EnteroBase.

number ID	Strain name (Biosample)	Collected by	Collection date	day	month	year	Geographic location	host	Host age	origin	isolation_source	Number of SNPs respect to 31/88 prophage	Strain name (EnteroBase)	ST	eBG	Serotype (Predicted)	aroC	dnaN	hemD	hisD	purE	sucA	thrA	Uberstrain	Bio Project ID	Sample ID	Secondary Sample ID
129	2009K-1726	CDC	2009		0	2009	USA:_Maryland	homo_sapiens	missing	human	missing	2	351865	1974	4	Enteritidis	5	2	378	7	6	6	11	SAL_GA3068AA	PRJNA168724	SAMN02937423	SRS666066
	ILBSalm5410214	missing	2008		0	2008	USA	missing	missing	unknown	missing	1	102_AM36958_B-sc-2013-02-27T10:27:08Z-1500	1974	4	Enteritidis	5	2	378	7	6	6	11	SAL_BA9071AA	PRJEB634	SAMEA2053458	ERS217249
	2009K-1733	CDC	missing			missing	USA	missing	missing	missing	missing	1	2009K-1733	1974	4	Enteritidis	5	2	378	7	6	6	11	SAL_BA1822AA	PRJNA230403	SAMN03857180	SRS994580
	2015AM-0832	CDC	May-2015		5	2015	USA	missing	80-89	human	Blood	1	2015AM-0832	1974	4	Enteritidis	5	2	378	7	6	6	11	SAL_KA1145AA	PRJNA230403	SAMN07281074	SRS2317104
	2015AM-1344	CDC	Jun-2015		6	2015	USA	missing	0-4	human	Stool	1	2015AM-1344	1974	4	Enteritidis	5	2	378	7	6	6	11	SAL_KA1146AA	PRJNA230403	SAMN07281076	SRS2317103
	2015AM-1371	CDC	missing			missing	USA	missing	missing	missing	missing	1	2015AM-1371	1974	4	Enteritidis	5	2	378	7	6	6	11	SAL_LA9892AA	PRJNA230403	SAMN07735252	SRS2564446
	2016K-0086	CDC	missing			missing	USA	missing	missing	missing	missing	1	2016K-0086	1974	4	Enteritidis	5	2	378	7	6	6	11	SAL_FA0845AA	PRJNA230403	SAMN04510731	SRS1307761
	2016K-0087	CDC	missing			missing	USA	missing	missing	missing	missing	1	2016K-0087	1974	4	Enteritidis	5	2	378	7	6	6	11	SAL_FA0846AA	PRJNA230403	SAMN04510730	SRS1307762
	CFSAN061128	CVM	missing			missing	USA:MD	missing	missing	food	Chicken_breast	1	CFSAN061128	1974	4	Enteritidis	5	2	378	7	6	6	11	SAL_JA5183AA	PRJNA186035	SAMN07146579	SRS2213516
	CVM_43837	CDC	2012			2012	USA	human	missing	human	clinical_sample	0	CVM 43837	1974	4	Enteritidis	5	2	378	7	6	6	11	SAL_JA4603AA	PRJNA242614	SAMN02912006	SRS2206833
	FDA00003429	FDA	2010-05-13	13	5	2010	Dominican_Republic	missing	missing	food	small_hot_peppers,_fresh	1	FDA00003429	1974	4	Enteritidis	5	2	378	7	6	6	11	SAL_GA8763AA	PRJNA186035	SAMN02846849	SRS1665470
	FDA00003430	FDA	2010-05-13	13	5	2010	Dominican_Republic	missing	missing	food	small_hot_peppers,_fresh	1	FDA00003430	1974	4	Enteritidis	5	2	378	7	6	6	11	SAL_GA8762AA	PRJNA186035	SAMN02846850	SRS1665471
	FDA00003431	FDA	2010-05-13	13	5	2010	Dominican_Republic	missing	missing	food	small_hot_peppers,_fresh	1	FDA00003431	1974	4	Enteritidis	5	2	378	7	6	6	11	SAL_GA8761AA	PRJNA186035	SAMN02846851	SRS1665472
	FDA00003987	FDA	2012-02-07	7	2	2012	Dominican_Republic	missing	missing	food	fresh_cucumber	1	FDA00003987	1974	4	Enteritidis	5	2	378	7	6	6	11	SAL_FA2315AA	PRJNA186035	SAMN02847404	SRS1353041
	FL_FLDACS-13767	FL	30-Jun-2011	30	6	2011	USA:FL	missing	missing	food	Peanut_Butter	1	FL_FLDACS-13767	1974	4	Enteritidis	5	2	378	7	6	6	11	SAL_EA0781AA	PRJNA183848	SAMN02253046	SRS503127
	FL_FLDACS-14008	FL	23-Jun-2011	23	6	2011	USA:FL	missing	missing	food	Peanut_Butter	1	FL_FLDACS-14008	1974	4	Enteritidis	5	2	378	7	6	6	11	SAL_EA1315AA	PRJNA183848	SAMN02253045	SRS500565
	FL_FLDACS-14009	FL	30-Jun-2011	30	6	2011	USA:FL	missing	missing	food	Peanut_Butter	1	FL_FLDACS-14009	1974	4	Enteritidis	5	2	378	7	6	6	11	SAL_EA0789AA	PRJNA183848	SAMN02253047	SRS503119
	FL_FLDACS-14012	FL	30-Jun-2011	30	6	2011	USA:FL	missing	missing	food	Peanut_Butter	1	FL_FLDACS-14012	1974	4	Enteritidis	5	2	378	7	6	6	11	SAL_EA0788AA	PRJNA183848	SAMN02253048	SRS503120
	FL_FLDACS-14013	FL	29-Feb-2012	29	2	2012	USA:FL	missing	missing	food	Peanut_Butter	1	FL_FLDACS-14013	1974	4	Enteritidis	5	2	378	7	6	6	11	SAL_EA0779AA	PRJNA183848	SAMN02253049	SRS503154
	FL_FLDACS-19514	FL	29-Feb-2012	29	2	2012	USA:FL	missing	missing	human	pet_food	1	FL_FLDACS-19514	1974	4	Enteritidis	5	2	378	7	6	6	11	SAL_EA0772AA	PRJNA183848	SAMN02253050	SRS503161
	FMA0080	FMA	2011-03-05	05	3	2011	Dominican_Republic	missing	missing	food	fresh_cucumber	1	FMA0080	1974	4	Enteritidis	5	2	378	7	6	6	11	SAL_DA9763AA	PRJNA186035	SAMN02345123	SRS554816
	FMA0090	FMA	2011-01-09	09	1	2011	Dominican_Republic	missing	missing	food	thai_chili_green_pepper	1	FMA0090	1974	4	Enteritidis	5	2	378	7	6	6	11	SAL_DA9347AA	PRJNA186035	SAMN02345068	SRS565875
	NY-30661	FDA	2003		0	2003	USA:MD	missing	missing	food	Chicken_breast	1	NY-30661	1974	4	Enteritidis	5	2	378	7	6	6	11	SAL_CA9303AA	PRJNA183850	SAMN02777712	SRS791458
	NY-swgs1260	NY	2014-04		4	2014	USA:NY	Homo_sapiens	missing	human	Stool	2	NY-swgs1260	1974	4	Enteritidis	5	2	378	7	6	6	11	SAL_DA7105AA	PRJNA227458	SAMN02742626	SRS620610
	PNUSAS001157	CDC	Dec-2010		12	2010	USA	missing	50-59	human	Stool	1	PNUSAS001157	1974	4	Enteritidis	5	2	378	7	6	6	11	SAL_EA5142AA	PRJNA230403	SAMN04229563	SRS1162936
	PNUSAS001158	CDC	Mar-2011		3	2011	USA	missing	30-39	human	Stool	3	PNUSAS001158	1974	4	Enteritidis	5	2	378	7	6	6	11	SAL_EA5141AA	PRJNA230403	SAMN04229564	SRS1162935
	PNUSAS001159	CDC	Mar-2011		3	2011	USA	missing	50-59	human	Blood	1	PNUSAS001159	1974	4	Enteritidis	5	2	378	7	6	6	11	SAL_EA5140AA	PRJNA230403	SAMN04229565	SRS1162932
	PNUSAS001160	CDC	Mar-2011		3	2011	USA	missing	0-4	human	Unknown	1	PNUSAS001160	1974	4	Enteritidis	5	2	378	7	6	6	11	SAL_EA5139AA	PRJNA230403	SAMN04229566	SRS1162926
	PNUSAS001161	CDC	Unknown			missing	USA	missing	10-19	human	Stool	1	PNUSAS001161	1974	4	Enteritidis	5	2	378	7	6	6	11	SAL_EA5138AA	PRJNA230403	SAMN04229567	SRS1162952
	PNUSAS001179	CDC	Apr-2011		4	2011	USA	missing	30-39	human	Stool	2	PNUSAS001179	1974	4	Enteritidis	5	2	378	7	6	6	11	SAL_EA5120AA	PRJNA230403	SAMN04252301	SRS1159352
	PNUSAS001180	CDC	Aug-2011		8	2011	USA	missing	10-19	human	Stool	1	PNUSAS001180	1974	4	Enteritidis	5	2	378	7	6	6	11	SAL_EA5119AA	PRJNA230403	SAMN04252302	SRS1159351
	PNUSAS001181	CDC	Sep-2011		9	2011	USA	missing	90-99	human	Stool	8	PNUSAS001181	1974	4	Enteritidis	5	2	378	7	6	6	11	SAL_EA5118AA	PRJNA230403	SAMN04252303	SRS1159350
	PNUSAS001182	CDC	Mar-2012		3	2012	USA	missing	60-69	human	Stool	3	PNUSAS001182	1974	4	Enteritidis	5	2	378	7	6	6	11	SAL_EA5117AA	PRJNA230403	SAMN04252304	SRS1159348
	PNUSAS001183	CDC	Apr-2012		4	2012	USA	missing	50-59	human	Stool	3	PNUSAS001183	1974	4	Enteritidis	5	2	378	7	6	6	11	SAL_EA5116AA	PRJNA230403	SAMN04252305	SRS1159344
	PNUSAS001184	CDC	Apr-2012		4	2012	USA	missing	40-49	human	Blood	1	PNUSAS001184	1974	4	Enteritidis	5	2	378	7	6	6	11	SAL_EA5115AA	PRJNA230403	SAMN04252306	SRS1159359
	PNUSAS001185	CDC	May-2012		5	2012	USA	missing	50-59	human	Blood	3	PNUSAS001185	1974	4	Enteritidis	5	2	378	7	6	6	11	SAL_EA5114AA	PRJNA230403	SAMN04252307	SRS1159358
	PNUSAS001186	CDC	May-2012		5	2012	USA	missing	60-69	human	Unknown	3	PNUSAS001186	1974	4	Enteritidis	5	2	378	7	6	6	11	SAL_EA5113AA	PRJNA230403	SAMN04252308	SRS1159357
	PNUSAS001214	CDC	Oct-2015		10	2015	USA	missing	0-4	human	Stool	2	PNUSAS001214	1974	4	Enteritidis	5	2	378	7	6	6	11	SAL_EA5306AA	PRJNA230403	SAMN04271279	SRS1171715
	PNUSAS001215	CDC	Oct-2015		10	2015	USA	missing	0-4	human	Urine	1	PNUSAS001215	1974	4	Enteritidis	5	2	378	7	6	6	11	SAL_EA5308AA	PRJNA230403	SAMN04271280	SRS1171717
	PNUSAS001216	CDC	Oct-2015		10	2015	USA	missing	40-49	human	Stool	2	PNUSAS001216	1974	4	Enteritidis	5	2	378	7	6	6	11	SAL_EA5309AA	PRJNA230403	SAMN04271281	SRS1171718
	PNUSAS001255	CDC	Jun-2012		6	2012	USA	missing	40-49	human	Stool	1	PNUSAS001255	1974	4	Enteritidis	5	2	378	7	6	6	11	SAL_EA6277AA	PRJNA230403	SAMN04304543	SRS1207357
	PNUSAS001256	CDC	Jul-2012		7	2012	USA	missing	40-49	human	Stool	1	PNUSAS001256	1974	4	Enteritidis	5	2	378	7	6	6	11	SAL_EA6276AA	PRJNA230403	SAMN04304544	SRS1207362
	PNUSAS001257	CDC	Jul-2012		7	2012	USA	missing	0-4	human	Stool	1	PNUSAS001257	1974	4	Enteritidis	5	2	378	7	6	6	11	SAL_EA6275AA	PRJNA230403	SAMN04304545	SRS1207361
	PNUSAS001258	CDC	Aug-2012		8	2012	USA	missing	40-49	human	Blood	1	PNUSAS001258	1974	4	Enteritidis	5	2	378	7	6	6	11	SAL_EA6274AA	PRJNA230403	SAMN04304546	SRS1207360
	PNUSAS001259	CDC	Nov-2012		11	2012	USA	missing	60-69	human	Rectal_Swab	3	PNUSAS001259	1974	4	Enteritidis	5	2	378	7	6	6	11	SAL_EA6273AA	PRJNA230403	SAMN04304547	SRS1207359
	PNUSAS001260	CDC	Unknown			missing	USA	missing	50-59	human	Stool	2	PNUSAS001260	1974	4	Enteritidis	5	2	378	7	6	6	11	SAL_EA6272AA	PRJNA230403	SAMN04304548	SRS1207358
	PNUSAS001261	CDC	Jan-2013		1	2013	USA	missing	0-4	human	Stool	1	PNUSAS001261	1974	4	Enteritidis	5	2	378	7	6	6	11	SAL_EA6271AA	PRJNA230403	SAMN04304549	SRS1207368
	PNUSAS001263	CDC	Mar-2013		3	2013	USA	missing	50-59	human	Blood	1	PNUSAS001263	1974	4	Enteritidis	5	2	378	7	6	6	11	SAL_EA6269AA	PRJNA230403	SAMN04304551	SRS1207366
	PNUSAS001282	CDC	Apr-2013		4	2013	USA	missing	40-49	human	Stool	1	PNUSAS001282	1974	4	Enteritidis	5	2	378	7	6	6	11	SAL_EA6321AA	PRJNA230403	SAMN04330112	SRS1207248
	PNUSAS001283	CDC	Unknown			missing	USA	missing	5-9	human	Stool	1	PNUSAS001283	1974	4	Enteritidis	5	2	378	7	6	6	11	SAL_EA6320AA	PRJNA230403	SAMN04330113	SRS1207247
	PNUSAS001284	CDC	May-2013		5	2013	USA	missing	20-29	human	Stool	1	PNUSAS001284	1974	4	Enteritidis	5	2	378	7	6	6	11	SAL_EA6319AA	PRJNA230403	SAMN04330114	SRS1207266
	PNUSAS001285	CDC	Jun-2013		6	2013	USA	missing	10-19	human	Stool	1	PNUSAS001285	1974	4	Enteritidis	5	2	378	7	6	6	11	SAL_EA6318AA	PRJNA230403	SAMN04330115	SRS1207265
	PNUSAS001286	CDC	Jun-2013		6	2013	USA	missing	30-39	human	Stool	1	PNUSAS001286	1974	4	Enteritidis	5	2	378	7	6	6	11	SAL_EA6317AA	PRJNA230403	SAMN04330116	SRS1207262
	PNUSAS001287	CDC	Aug-2013		8	2013	USA	missing	50-59	human	Stool	8	PNUSAS001287	1974	4	Enteritidis	5	2	378	7	6	6	11	SAL_EA6316AA	PRJNA230403	SAMN04330117	SRS1207263
	PNUSAS001288	CDC	Oct-2013		10	2013	USA	missing	50-59	human	Stool	2	PNUSAS001288	1974	4	Enteritidis	5	2	378	7	6	6	11	SAL_EA6315AA	PRJNA230403	SAMN04330118	SRS1207270
	PNUSAS001289	CDC	Oct-2013		10	2013	USA	missing	10-19	human	Stool	1	PNUSAS001289	1974	4	Enteritidis	5	2	378	7	6	6	11	SAL_EA6314AA	PRJNA230403	SAMN04330119	SRS1207269
	PNUSAS001290	CDC	Oct-2013		10	2013	USA	missing	30-39	human	Stool	1	PNUSAS001290	1974	4	Enteritidis	5	2	378	7	6	6	11	SAL_EA6313AA	PRJNA230403	SAMN04330120	SRS1207268
	PNUSAS001291	CDC	Nov-2013		11	2013	USA	missing	5-9	human	Stool	2	PNUSAS001291	1974	4	Enteritidis	5	2	378	7	6	6	11	SAL_EA6312AA	PRJNA230403	SAMN04330121	SRS1207273
128	PNUSAS001292	CDC	Nov-2013		11	2013	USA	missing	50-59	human	Other – clinical	1	PNUSAS001292	1974	4	Enteritidis	5	2	378	7	6	6	11	SAL_EA6311AA	PRJNA230403	SAMN04330122	SRS1207272
127	PNUSAS001293	CDC	Jan-2014		1	2014	USA	missing	40-49	human	Stool	1	PNUSAS001293	1974	4	Enteritidis	5	2	378	7	6	6	11	SAL_EA6310AA	PRJNA230403	SAMN04330123	SRS1207271
	PNUSAS001294	CDC	Feb-2014		2	2014	USA	missing	50-59	human	Blood	1	PNUSAS001294	1974	4	Enteritidis	5	2	378	7	6	6	11	SAL_EA6309AA	PRJNA230403	SAMN04330124	SRS1207292
	PNUSAS001295	CDC	Feb-2014		2	2014	USA	missing	Unknown	human	Stool	3	PNUSAS001295	1974	4	Enteritidis	5	2	378	7	6	6	11	SAL_EA6308AA	PRJNA230403	SAMN04330125	SRS1207291
126	PNUSAS001296	CDC	Feb-2014		2	2014	USA	missing	0-4	human	Urine	3	PNUSAS001296	1974	4	Enteritidis	5	2	378	7	6	6	11	SAL_EA6307AA	PRJNA230403	SAMN04330126	SRS1207290
	PNUSAS001317	CDC	Jun-2014		6	2014	USA	missing	30-39	human	Stool	1	PNUSAS001317	1974	4	Enteritidis	5	2	378	7	6	6	11	SAL_EA6594AA	PRJNA230403	SAMN04339744	SRS1212723
	PNUSAS001318	CDC	Aug-2014		8	2014	USA	missing	40-49	human	Stool	1	PNUSAS001318	1974	4	Enteritidis	5	2	378	7	6	6	11	SAL_EA6593AA	PRJNA230403	SAMN04339745	SRS1212722
	PNUSAS001375	CDC	Oct-2015		10	2015	USA	missing	0-4	human	Stool	1	PNUSAS001375	1974	4	Enteritidis	5	2	378	7	6	6	11	SAL_EA8880AA	PRJNA230403	SAMN04407121	SRS1249559
	PNUSAS001440	CDC	Sep-2015		9	2015	USA	missing	80-89	human	Blood	1	PNUSAS001440	1974	4	Enteritidis	5	2	378	7	6	6	11	SAL_EA9392AA	PRJNA230403	SAMN04442309	SRS1271236
	PNUSAS001441	CDC	Oct-2015		10	2015	USA	missing	30-39	human	Stool	2	PNUSAS001441	1974	4	Enteritidis	5	2	378	7	6	6	11	SAL_EA9391AA	PRJNA230403	SAMN04442310	SRS1271235
	PNUSAS001442	CDC	Sep-2015		9	2015	USA	missing	40-49	human	Stool	1	PNUSAS001442	1974	4	Enteritidis	5	2	378	7	6	6	11	SAL_EA9390AA	PRJNA230403	SAMN04442311	SRS1271247
	PNUSAS001443	CDC	Sep-2015		9	2015	USA	missing	Unknown	human	Stool	1	PNUSAS001443	1974	4	Enteritidis	5	2	378	7	6	6	11	SAL_EA9389AA	PRJNA230403	SAMN04442312	SRS1271257
	PNUSAS001444	CDC	Oct-2015		10	2015	USA	missing	0-4	human	Stool	1	PNUSAS001444	1974	4	Enteritidis	5	2	378	7	6	6	11	SAL_EA9388AA	PRJNA230403	SAMN04442313	SRS1271267
	PNUSAS001446	CDC	Oct-2015		10	2015	USA	missing	5-9	human	Stool	2	PNUSAS001446	1974	4	Enteritidis	5	2	378	7	6	6	11	SAL_EA9386AA	PRJNA230403	SAMN04442315	SRS1271271
	PNUSAS001447	CDC	Oct-2015		10	2015	USA	missing	5-9	human	Stool	1	PNUSAS001447	1974	4	Enteritidis	5	2	378	7	6	6	11	SAL_EA9385AA	PRJNA230403	SAMN04442316	SRS1271294
	PNUSAS001448	CDC	Nov-2015		11	2015	USA	missing	0-4	human	Stool	1	PNUSAS001448	1974	4	Enteritidis	5	2	378	7	6	6	11	SAL_EA9384AA	PRJNA230403	SAMN04442317	SRS1271293
	PNUSAS001526	CDC	Jan-2016		1	2016	USA	missing	Unknown	human	Stool	8	PNUSAS001526	1974	4	Enteritidis	5	2	378	7	6	6	11	SAL_FA1097AA	PRJNA230403	SAMN04490806	SRS1308369
	PNUSAS001728	CDC	Feb-2016		2	2016	USA	missing	0-4	human	Stool	9	PNUSAS001728	1974	4	Enteritidis	5	2	378	7	6	6	11	SAL_FA2425AA	PRJNA230403	SAMN04546470	SRS1353915
	PNUSAS001752	CDC	Feb-2016		2	2016	USA	missing	0-4	human	Stool	1	PNUSAS001752	1974	4	Enteritidis	5	2	378	7	6	6	11	SAL_FA2415AA	PRJNA230403	SAMN04558120	SRS1353923
	PNUSAS001819	CDC	Mar-2016		3	2016	USA	missing	0-4	human	Stool	2	PNUSAS001819	1974	4	Enteritidis	5	2	378	7	6	6	11	SAL_FA5559AA	PRJNA230403	SAMN04590155	SRS1404918
	PNUSAS001823	CDC	Mar-2016		3	2016	USA	missing	0-4	human	Stool	2	PNUSAS001823	1974	4	Enteritidis	5	2	378	7	6	6	11	SAL_FA5556AA	PRJNA230403	SAMN04590159	SRS1404920
	PNUSAS001825	CDC	Unknown			missing	USA	missing	0-4	human	Stool	1	PNUSAS001825	1974	4	Enteritidis	5	2	378	7	6	6	11	SAL_FA5554AA	PRJNA230403	SAMN04590161	SRS1404925
	PNUSAS002044	CDC	Unknown			missing	USA	missing	Unknown	human	Stool	1	PNUSAS002044	1974	4	Enteritidis	5	2	378	7	6	6	11	SAL_FA6123AA	PRJNA230403	SAMN04902836	SRS1420826
	PNUSAS002098	CDC	Mar-2016		3	2016	USA	missing	10-19	human	Stool	9	PNUSAS002098	1974	4	Enteritidis	5	2	378	7	6	6	11	SAL_FA6037AA	PRJNA230403	SAMN04925737	SRS1421836
	PNUSAS002123	CDC	Apr-2016		4	2016	USA	missing	30-39	human	Stool	2	PNUSAS002123	1974	4	Enteritidis	5	2	378	7	6	6	11	SAL_FA6615AA	PRJNA230403	SAMN04961833	SRS1434151
	PNUSAS002196	missing	Jan-2016		1	2016	USA	missing	30-39	human	Stool	1	PNUSAS002196	1974	4	Enteritidis	5	2	378	7	6	6	11	SAL_FA7148AA	PRJNA230403	SAMN05004759	SRS1462752
	PNUSAS002451	CDC	May-2016		5	2016	USA	missing	5-9	human	Stool	2	PNUSAS002451	1974	4	Enteritidis	5	2	378	7	6	6	11	SAL_FA7950AA	PRJNA230403	SAMN05195132	SRS1504615
	PNUSAS002498	CDC	May-2016		5	2016	USA	missing	60-69	human	Stool	8	PNUSAS002498	1974	4	Enteritidis	5	2	378	7	6	6	11	SAL_FA8409AA	PRJNA230403	SAMN05210274	SRS1523103
	PNUSAS002499	CDC	May-2016		5	2016	USA	missing	40-49	human	Stool	1	PNUSAS002499	1974	4	Enteritidis	5	2	378	7	6	6	11	SAL_FA8408AA	PRJNA230403	SAMN05210275	SRS1523104
	PNUSAS002580	CDC	May-2016		5	2016	USA	missing	20-29	human	Stool	8	PNUSAS002580	1974	4	Enteritidis	5	2	378	7	6	6	11	SAL_GA5493AA	PRJNA230403	SAMN05271601	SRS1572800
	PNUSAS002664	CDC	May-2016		5	2016	USA	missing	60-69	human	Stool	8	PNUSAS002664	1974	4	Enteritidis	5	2	378	7	6	6	11	SAL_GA4342AA	PRJNA230403	SAMN05294445	SRS1531532
	PNUSAS003171	CDC	Jul-2016		7	2016	USA	missing	0-4	human	Stool	1	PNUSAS003171	1974	4	Enteritidis	5	2	378	7	6	6	11	SAL_GA6571AA	PRJNA230403	SAMN05504512	SRS1601227
	PNUSAS003174	CDC	Jul-2016		7	2016	USA	missing	0-4	human	Stool	1	PNUSAS003174	1974	4	Enteritidis	5	2	378	7	6	6	11	SAL_GA6568AA	PRJNA230403	SAMN05504515	SRS1601245
	PNUSAS003232	CDC	Jul-2016		7	2016	USA	missing	30-39	human	Stool	3	PNUSAS003232	1974	4	Enteritidis	5	2	378	7	6	6	11	SAL_GA7477AA	PRJNA230403	SAMN05509868	SRS1625279
	PNUSAS003351	CDC	Jul-2016		7	2016	USA	missing	0-4	human	Stool	3	PNUSAS003351	1974	4	Enteritidis	5	2	378	7	6	6	11	SAL_GA8103AA	PRJNA230403	SAMN05559569	SRS1631502
	PNUSAS003513	CDC	Jul-2016		7	2016	USA	missing	0-4	human	Stool	1	PNUSAS003513	1974	4	Enteritidis	5	2	378	7	6	6	11	SAL_GA8645AA	PRJNA230403	SAMN05603494	SRS1659798
	PNUSAS003514	CDC	Jul-2016		7	2016	USA	missing	0-4	human	Stool	2	PNUSAS003514	1974	4	Enteritidis	5	2	378	7	6	6	11	SAL_GA8644AA	PRJNA230403	SAMN05603495	SRS1659804
	PNUSAS003636	CDC	Jul-2016		7	2016	USA	missing	40-49	human	Stool	1	PNUSAS003636	1974	4	Enteritidis	5	2	378	7	6	6	11	SAL_GA8920AA	PRJNA230403	SAMN05713999	SRS1677071
	PNUSAS003855	CDC	Aug-2016		8	2016	USA	missing	70-79	human	Urine	1	PNUSAS003855	1974	4	Enteritidis	5	2	378	7	6	6	11	SAL_GA9424AA	PRJNA230403	SAMN05762919	SRS1704594
	PNUSAS004246	CDC	Aug-2016		8	2016	USA	missing	20-29	human	Stool	8	PNUSAS004246	1974	4	Enteritidis	5	2	378	7	6	6	11	SAL_HA0899AA	PRJNA230403	SAMN05851343	SRS1738713
	PNUSAS004260	CDC	Aug-2016		8	2016	USA	missing	0-4	human	Stool	3	PNUSAS004260	1974	4	Enteritidis	5	2	378	7	6	6	11	SAL_HA0885AA	PRJNA230403	SAMN05851356	SRS1738735
	PNUSAS004270	CDC	Aug-2016		8	2016	USA	missing	30-39	human	Stool	8	PNUSAS004270	1974	4	Enteritidis	5	2	378	7	6	6	11	SAL_HA0908AA	PRJNA230403	SAMN05851365	SRS1738704
	PNUSAS004702	CDC	Aug-2016		8	2016	USA	missing	0-4	human	Stool	1	PNUSAS004702	1974	4	Enteritidis	5	2	378	7	6	6	11	SAL_HA3188AA	PRJNA230403	SAMN05943269	SRS1789895
	PNUSAS005243	CDC	Sep-2016		9	2016	USA	missing	0-4	human	Stool	1	PNUSAS005243	1974	4	Enteritidis	5	2	378	7	6	6	11	SAL_IA4615AA	PRJNA230403	SAMN06049079	SRS1954122
	PNUSAS005845	CDC	Sep-2016		9	2016	USA	missing	30-39	human	Stool	2	PNUSAS005845	1974	4	Enteritidis	5	2	378	7	6	6	11	SAL_HA7399AA	PRJNA230403	SAMN06140365	SRS1867883
	PNUSAS005862	CDC	Sep-2016		9	2016	USA	missing	0-4	human	Stool	1	PNUSAS005862	1974	4	Enteritidis	5	2	378	7	6	6	11	SAL_HA7386AA	PRJNA230403	SAMN06140382	SRS1867919
	PNUSAS006823	CDC	Nov-2016		11	2016	USA	missing	60-69	human	Blood	1	PNUSAS006823	1974	4	Enteritidis	5	2	378	7	6	6	11	SAL_IA0534AA	PRJNA230403	SAMN06215598	SRS1929455
	PNUSAS006833	CDC	Nov-2016		11	2016	USA	missing	10-19	human	Stool	8	PNUSAS006833	1974	4	Enteritidis	5	2	378	7	6	6	11	SAL_IA0244AA	PRJNA230403	SAMN06215586	SRS1926859
	PNUSAS007402	CDC	Nov-2016		11	2016	USA	missing	0-4	human	Stool	2	PNUSAS007402	1974	4	Enteritidis	5	2	378	7	6	6	11	SAL_IA0520AA	PRJNA230403	SAMN06236492	SRS1929469
	PNUSAS007403	CDC	Nov-2016		11	2016	USA	missing	40-49	human	Blood	1	PNUSAS007403	1974	4	Enteritidis	5	2	378	7	6	6	11	SAL_IA0518AA	PRJNA230403	SAMN06236493	SRS1929471
	PNUSAS007776	CDC	Dec-2016		12	2016	USA	missing	0-4	human	Stool	9	PNUSAS007776	1974	4	Enteritidis	5	2	378	7	6	6	11	SAL_IA6173AA	PRJNA230403	SAMN06285361	SRS1964071
	PNUSAS008029	CDC	Oct-2016		10	2016	USA	missing	20-29	human	Stool	1	PNUSAS008029	1974	4	Enteritidis	5	2	378	7	6	6	11	SAL_IA5677AA	PRJNA230403	SAMN06310377	SRS1972839
	PNUSAS008320	CDC	missing			missing	USA	missing	missing	missing	missing	3	PNUSAS008320	1974	4	Enteritidis	5	2	378	7	6	6	11	SAL_IA6487AA	PRJNA230403	SAMN06335590	SRS1989719
	PNUSAS008942	CDC	missing			missing	USA	missing	missing	missing	missing	1	PNUSAS008942	1974	4	Enteritidis	5	2	378	7	6	6	11	SAL_IA9443AA	PRJNA230403	SAMN06462340	SRS2060079
	PNUSAS009638	CDC	Feb-2017		2	2017	USA	missing	0-4	human	Stool	1	PNUSAS009638	1974	4	Enteritidis	5	2	378	7	6	6	11	SAL_IA9505AA	PRJNA230403	SAMN06547084	SRS2060017
	PNUSAS009643	CDC	Feb-2017		2	2017	USA	missing	0-4	human	Stool	8	PNUSAS009643	1974	4	Enteritidis	5	2	378	7	6	6	11	SAL_KA2941AA	PRJNA230403	SAMN06547089	SRS2339605
	PNUSAS009908	CDC	Feb-2017		2	2017	USA	missing	20-29	human	Stool	3	PNUSAS009908	1974	4	Enteritidis	5	2	378	7	6	6	11	SAL_IA9583AA	PRJNA230403	SAMN06606924	SRS2062211
	PNUSAS009911	CDC	Feb-2017		2	2017	USA	missing	0-4	human	Stool	2	PNUSAS009911	1974	4	Enteritidis	5	2	378	7	6	6	11	SAL_IA9577AA	PRJNA230403	SAMN06606927	SRS2062218
	PNUSAS009914	CDC	Feb-2017		2	2017	USA	missing	60-69	human	Stool	2	PNUSAS009914	1974	4	Enteritidis	5	2	378	7	6	6	11	SAL_IA9573AA	PRJNA230403	SAMN06606930	SRS2062222
	PNUSAS010346	CDC	Feb-2017		2	2017	USA	missing	0-4	human	Blood	1	PNUSAS010346	1974	4	Enteritidis	5	2	378	7	6	6	11	SAL_JA1518AA	PRJNA230403	SAMN06689009	SRS2110300
	PNUSAS010559	CDC	Mar-2017		3	2017	USA	missing	40-49	human	Stool	3	PNUSAS010559	1974	4	Enteritidis	5	2	378	7	6	6	11	SAL_JA1511AA	PRJNA230403	SAMN06689011	SRS2110337
	PNUSAS010562	CDC	missing			missing	USA	missing	missing	missing	missing	2	PNUSAS010562	1974	4	Enteritidis	5	2	378	7	6	6	11	SAL_KA3428AA	PRJNA230403	SAMN06645629	SRS2349196
	PNUSAS010563	CDC	Mar-2017		3	2017	USA	missing	0-4	human	Stool	1	PNUSAS010563	1974	4	Enteritidis	5	2	378	7	6	6	11	SAL_JA1104AA	PRJNA230403	SAMN06645630	SRS2102124
	PNUSAS010564	CDC	Mar-2017		3	2017	USA	missing	70-79	human	Blood	2	PNUSAS010564	1974	4	Enteritidis	5	2	378	7	6	6	11	SAL_JA1235AA	PRJNA230403	SAMN06645631	SRS2098888
	PNUSAS010741	CDC	Mar-2017		3	2017	USA	missing	40-49	human	Stool	1	PNUSAS010741	1974	4	Enteritidis	5	2	378	7	6	6	11	SAL_JA0502AA	PRJNA230403	SAMN06645434	SRS2079459
	PNUSAS011201	CDC	missing			missing	USA	missing	missing	missing	missing	10	PNUSAS011201	1974	4	Enteritidis	5	2	378	7	6	6	11	SAL_JA1792AA	PRJNA230403	SAMN06689023	SRS2115215
	PNUSAS011773	CDC	missing			missing	USA	missing	missing	missing	missing	1	PNUSAS011773	1974	4	Enteritidis	5	2	378	7	6	6	11	SAL_JA2396AA	PRJNA230403	SAMN06758406	SRS2135006
	PNUSAS011781	CDC	missing			missing	USA	missing	missing	missing	missing	1	PNUSAS011781	1974	4	Enteritidis	5	2	378	7	6	6	11	SAL_JA2390AA	PRJNA230403	SAMN06758413	SRS2135013
	PNUSAS014648	CDC	May-2017		5	2016	USA	missing	20-29	human	Stool	2	PNUSAS014648	1974	4	Enteritidis	5	2	378	7	6	6	11	SAL_KA2697AA	PRJNA230403	SAMN07184666	SRS2342633
	PNUSAS015758	CDC	missing			missing	USA	missing	missing	missing	missing	1	PNUSAS015758	1974	4	Enteritidis	5	2	378	7	6	6	11	SAL_KA2685AA	PRJNA230403	SAMN07209697	SRS2342645
	PNUSAS015759	CDC	missing			missing	USA	missing	missing	missing	missing	2	PNUSAS015759	1974	4	Enteritidis	5	2	378	7	6	6	11	SAL_KA2690AA	PRJNA230403	SAMN07209698	SRS2342640
	PNUSAS015894	CDC	missing			missing	USA	missing	missing	missing	missing	1	PNUSAS015894	1974	4	Enteritidis	5	2	378	7	6	6	11	SAL_KA0156AA	PRJNA230403	SAMN07224016	SRS2296841
	PNUSAS016130	CDC	missing			missing	USA	missing	missing	missing	missing	2	PNUSAS016130	1974	4	Enteritidis	5	2	378	7	6	6	11	SAL_KA2677AA	PRJNA230403	SAMN07270958	SRS2342652
	PNUSAS016134	CDC	Dec-2016		12	2016	USA	Homo_sapiens	40-49	human	Stool	2	PNUSAS016134	1974	4	Enteritidis	5	2	378	7	6	6	11	SAL_KA0353AA	PRJNA230403	SAMN07227124	SRS2308465
	PNUSAS016480	CDC	missing			missing	USA	missing	missing	missing	missing	8	PNUSAS016480	1974	4	Enteritidis	5	2	378	7	6	6	11	SAL_KA2661AA	PRJNA230403	SAMN07270965	SRS2342670
	PNUSAS016995	CDC	missing			missing	USA	missing	missing	missing	missing	1	PNUSAS016995	1974	4	Enteritidis	5	2	378	7	6	6	11	SAL_KA3642AA	PRJNA230403	SAMN07279069	SRS2352215
	PNUSAS017244	CDC	missing			missing	USA	missing	missing	missing	missing	1	PNUSAS017244	1974	4	Enteritidis	5	2	378	7	6	6	11	SAL_KA5964AA	PRJNA230403	SAMN07373197	SRS2391511
	PNUSAS017604	CDC	missing			missing	USA	missing	missing	missing	missing	3	PNUSAS017604	1974	4	Enteritidis	5	2	378	7	6	6	11	SAL_KA3631AA	PRJNA230403	SAMN07341871	SRS2352227
	PNUSAS017612	CDC	missing			missing	USA	missing	missing	missing	missing	1	PNUSAS017612	1974	4	Enteritidis	5	2	378	7	6	6	11	SAL_KA3619AA	PRJNA230403	SAMN07341879	SRS2352239
	PNUSAS018393	CDC	missing			missing	USA	missing	missing	missing	missing	1	PNUSAS018393	1974	4	Enteritidis	5	2	378	7	6	6	11	SAL_KA6955AA	PRJNA230403	SAMN07373215	SRS2400255
	PNUSAS018395	CDC	missing			missing	USA	missing	missing	missing	missing	8	PNUSAS018395	1974	4	Enteritidis	5	2	378	7	6	6	11	SAL_KA6940AA	PRJNA230403	SAMN07373217	SRS2400239
	PNUSAS019648	missing	Jan-2017		1	2017	USA	missing	70-79	human	Stool	0	PNUSAS019648	1974	4	Enteritidis	5	2	378	7	6	6	11	SAL_KA8552AA	PRJNA230403	SAMN07483872	SRS2432618
	PNUSAS019678	CDC	missing			missing	USA	missing	missing	missing	missing	1	PNUSAS019678	1974	4	Enteritidis	5	2	378	7	6	6	11	SAL_KA7503AA	PRJNA230403	SAMN07428707	SRS2414039
	PNUSAS019680	CDC	missing			missing	USA	missing	missing	missing	missing	2	PNUSAS019680	1974	4	Enteritidis	5	2	378	7	6	6	11	SAL_KA7505AA	PRJNA230403	SAMN07428709	SRS2414040
	PNUSAS019682	CDC	missing			missing	USA	missing	missing	missing	missing	1	PNUSAS019682	1974	4	Enteritidis	5	2	378	7	6	6	11	SAL_KA7497AA	PRJNA230403	SAMN07428711	SRS2414086
	PNUSAS019873	CDC	missing			missing	USA	missing	missing	missing	missing	1	PNUSAS019873	1974	4	Enteritidis	5	2	378	7	6	6	11	SAL_KA7865AA	PRJNA230403	SAMN07456572	SRS2420806
	PNUSAS020638	missing	Jun-2016		6	2016	USA	missing	40-49	human	Stool	2	PNUSAS020638	1974	4	Enteritidis	5	2	378	7	6	6	11	SAL_KA9674AA	PRJNA230403	SAMN07504913	SRS2441820
	PNUSAS020639	missing	Jun-2016		6	2016	USA	missing	70-79	human	Stool	2	PNUSAS020639	1974	4	Enteritidis	5	2	378	7	6	6	11	SAL_KA9672AA	PRJNA230403	SAMN07504914	SRS2441822
	PNUSAS020939	CDC	missing			missing	USA	missing	missing	missing	missing	2	PNUSAS020939	1974	4	Enteritidis	5	2	378	7	6	6	11	SAL_LA0710AA	PRJNA230403	SAMN07554668	SRS2476748
	PNUSAS021405	CDC	missing			missing	USA	missing	missing	missing	missing	1	PNUSAS021405	1974	4	Enteritidis	5	2	378	7	6	6	11	SAL_LA0477AA	PRJNA230403	SAMN07537074	SRS2467837
	PNUSAS021407	CDC	missing			missing	USA	missing	missing	missing	missing	1	PNUSAS021407	1974	4	Enteritidis	5	2	378	7	6	6	11	SAL_LA0486AA	PRJNA230403	SAMN07537076	SRS2468032
	PNUSAS021720	CDC	missing			missing	USA	missing	missing	missing	missing	1	PNUSAS021720	1974	4	Enteritidis	5	2	378	7	6	6	11	SAL_LA1344AA	PRJNA230403	SAMN07559266	SRS2478644
	PNUSAS021725	CDC	missing			missing	USA	missing	missing	missing	missing	1	PNUSAS021725	1974	4	Enteritidis	5	2	378	7	6	6	11	SAL_LA1250AA	PRJNA230403	SAMN07559271	SRS2480526
	PNUSAS021930	CDC	missing			missing	USA	missing	missing	missing	missing	1	PNUSAS021930	1974	4	Enteritidis	5	2	378	7	6	6	11	SAL_LA3805AA	PRJNA230403	SAMN07595166	SRS2506147
	PNUSAS023210	CDC	missing			missing	USA	missing	missing	missing	missing	2	PNUSAS023210	1974	4	Enteritidis	5	2	378	7	6	6	11	SAL_LA6743AA	PRJNA230403	SAMN07666361	SRS2546927
	PNUSAS024035	missing	missing			missing	USA	missing	missing	missing	missing	2	PNUSAS024035	1974	4	Enteritidis	5	2	378	7	6	6	11	SAL_LA6470AA	PRJNA230403	SAMN07694679	SRS2547258

The metadata showed here was obtained from both EnteroBase and BioSample databases (see color key below). The first column contains the number ID of those strains present in table S1. Also, the number of SNPs obtained by comparison of the 31/88 ST-1974 prophage sequence with the contigs sequences of each strain containing the ST-1974 prophage is showed (column header highlighted in gray).

Color Key

(green)	Data obtained from BioSample database
(cyan)	Data obtained from EnteroBase

Figure S1. ST-1974 prophage diversity.

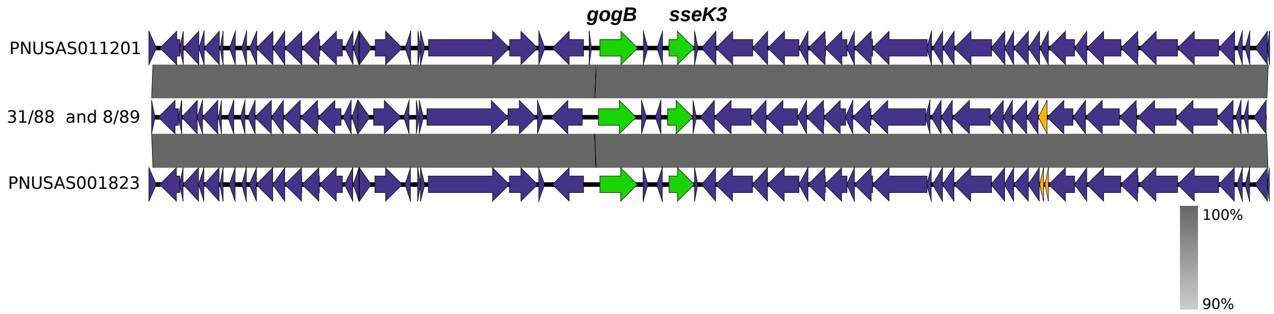

This figure shows the comparison between the ST-1974 prophage of strains 31/88 and 8/89 and the two most diverse sequences found in the ST-1974 genomes from EnteroBase. The upper line represent the sequence of the corresponding prophage in strain PNUSAS0011201, with a 99.98 % identity respect to the 31/88-8/89 prophage. The bottom line represent the prophage sequence in strain PNUSAS001823, that harbor an early stop codon in one of the prophage genes (highlighted in orange in the figure, corresponding to the CDS 50 in Table S3). *gogB* and *sseK3* coding sequences are highlighted in green.

Figure S2: Phylogenetic tree of representative *S*. Enteritidis genomes showing bootstrap values at the nodes.

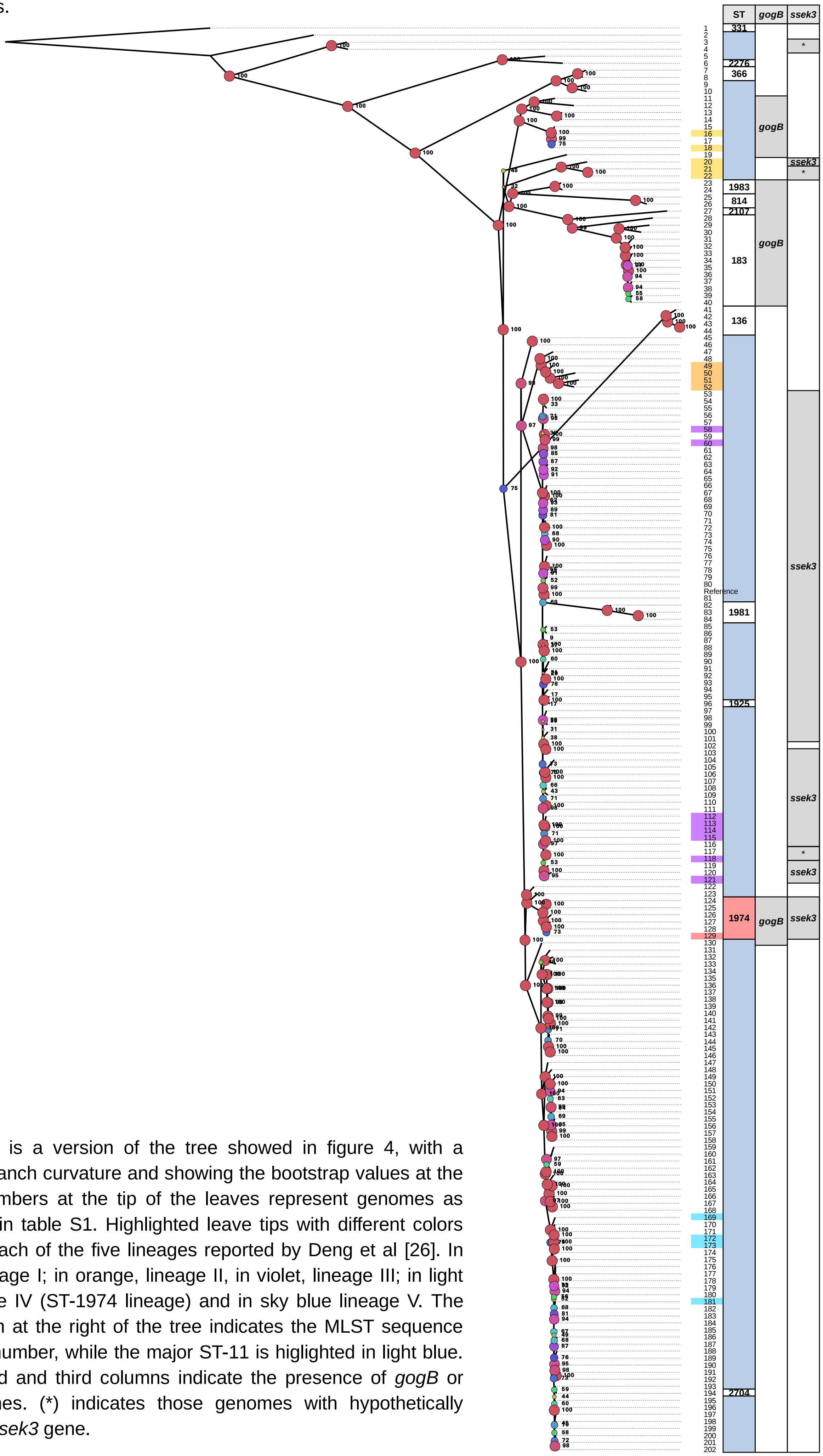

This figure is a version of the tree showed in figure 4, with a different branch curvature and showing the bootstrap values at the nodes. Numbers at the tip of the leaves represent genomes as numbered in table S1. Highlighted leave tips with different colors indicates each of the five lineages reported by Deng et al [26]. In yellow, lineage I; in orange, lineage II, in violet, lineage III; in light red, lineage IV (ST-1974 lineage) and in sky blue lineage V. The first column at the right of the tree indicates the MLST sequence type (ST) number, while the major ST-11 is higlighted in light blue. The second and third columns indicate the presence of *gogB* or *sseK3* genes. (*) indicates those genomes with hypothetically disrupted *ssek3* gene.
